# Smenamide A Analogues. Synthesis and Biological Activity on Multiple Myeloma Cells

**DOI:** 10.3390/md16060206

**Published:** 2018-06-13

**Authors:** Alessia Caso, Ilaria Laurenzana, Daniela Lamorte, Stefania Trino, Germana Esposito, Vincenzo Piccialli, Valeria Costantino

**Affiliations:** 1 Department of Pharmacy, University of Naples Federico II, 80131 Napoli, Italy; alessia.caso@unina.it (A.C.); germana.esposito@unina.it (G.E.); 2 Laboratory of Pre-Clinical and Translational Research, IRCCS—Referral Cancer Center of Basilicata (CROB), 85028 Rionero in Vulture, Italy; ilaria.laurenzana@crob.it (I.L.); daniela.lamorte@crob.it (D.L.); stefania.trino@crob.it (S.T.); 3 Department of Chemical Sciences, University of Naples Federico II, via Cintia 4, 80126 Naples, Italy

**Keywords:** smenamides, marine natural products, peptide/polyketide molecules, synthetic analogues, functional-analogues, antiproliferative activity, MM cell line

## Abstract

Smenamides are an intriguing class of peptide/polyketide molecules of marine origin showing antiproliferative activity against lung cancer Calu-1 cells at nanomolar concentrations through a clear pro-apoptotic mechanism. To probe the role of the activity-determining structural features, the 16-*epi*-analogue of smenamide A and eight simplified analogues in the 16-*epi* series were prepared using a flexible synthetic route. The synthetic analogues were tested on multiple myeloma (MM) cell lines showing that the configuration at C-16 slightly affects the activity, since the 16-*epi*-derivative is still active at nanomolar concentrations. Interestingly, it was found that the truncated compound **8**, mainly composed of the pyrrolinone terminus, was not active, while compound **13**, essentially lacking the pyrrolinone moiety, was 1000-fold less active than the intact substance and was the most active among all the synthesized compounds.

## 1. Introduction

Marine sponges, together with their symbiotic microorganisms, have proven to be a rich source of skeletally new substances [1,2,3], which have often inspired novel strategies in anticancer drug discovery. Targeted cancer therapies consist of “drugs” which interfere with specific molecules necessary for tumor growth and progression. A primary goal of these therapies is to fight cancer cells with more precision without hitting normal cells. These drugs are classified into monoclonal antibodies, directed against antigens expressed on the neoplastic cell surface, and small molecules, usually designed to interfere with protein targets [4].

**Figure 1:**
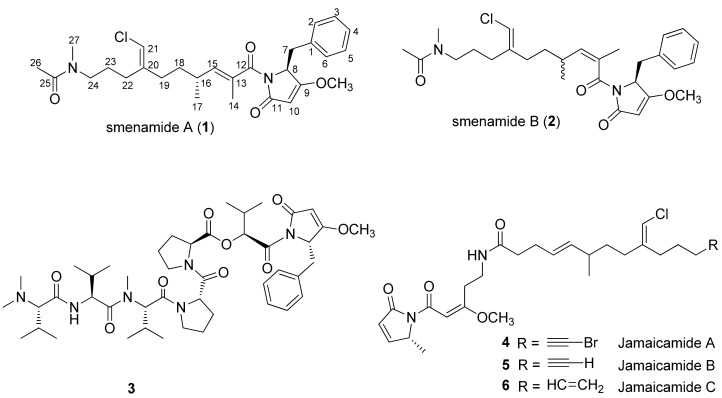
Smenamide A (**1**) and B (**2**), dolastatin-15 (**3**), and jamaicamides (**4**–**6**). Configuration at C-16 in smenamide A as determined by synthesis [8].

Smenamides A (**1**) and B (**2**) (Figure 1) are highly functionalized peptide/polyketide substances isolated by our group in 2013 from the Caribbean sponge *Smenospongia aurea* [5]. They have proven to be interesting for their structural features, such as the unusual *N*-methylacetamide western terminus, the dolapyrrolidone eastern terminus, typical of dolastatin-15 (**3**), a potent antimitotic agent derived from *Dolabella auricularia* [6], and the chlorovinyl functional group, common to some cyanobacterial metabolites, such as jamaicamides (**4**–**6**, Figure 1), isolated from *Lyngbiamajuscula* [7]. The only difference between the two smenamides resides in the configuration of the C-13/C-15 double bond positioned close to the middle part of the polyketide portion of the molecule. It has been speculated that this could determine a different overall shape and, as a consequence, the different biological behavior observed for smenamides [5].

**Figure 2:**
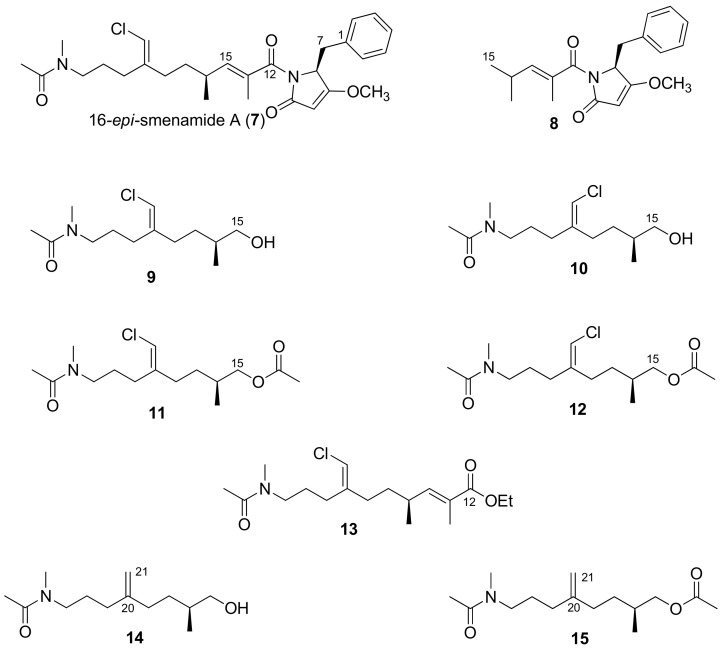
16-*epi*-smenamide A (**7**) and its analogues **8**–**15**. For structural comparison, numeration of analogues is in agreement with that of 16-*epi*-smenamide A.

Smenamides have proven to be active in blocking the proliferation of the Calu-1 cancer cell line at nanomolar concentrations, working, however, with a different mechanism of action. Smenamide A, more interestingly, acts with a clear pre-apoptotic mechanism proving to be the more promising as a lead compound. It is worth stating that the configuration at C-16 in both smenamides remained unassigned in the original study due to the limited amount of the natural substances available. In a recent study, a chiral protocol strategy aimed at the total synthesis of the smenamide family was designed, starting from commercially available *S*-citronellene, a cheap starting material [8]. Two stereoisomers of smenamide A, namely *ent*-smenamide A and 16-*epi*-smenamide A (**7**, Figure 2), were synthesized. This synthetic effort allowed us to determine the C-16 configuration of smenamide A as *R* (Figure 1), as well as to develop a flexible synthetic route towards this class of substances.

In the present study, the antiproliferative activity of 16-*epi*-smenamide A has been evaluated on multiple myeloma (MM) cell lines. MM is a clonal plasma cell malignancy accounting for approximately 13% of all hematological cancers [9]. It originates from post-germinal centre B cells that accumulate somatic hypermutation and immunoglobulin heavy-chain class switching [8]. Several novel agents have been introduced into clinical practice but, after an initial response, most patients relapse or progress with a treatment-refractory disease [10]. For this reason, MM still proves to be incurable for most patients. In this scenario, it is necessary to develop new agents targeting novel pathways relevant for the MM cells, thereby increasing the range of available therapies.

In addition to 16-*epi*-smenamide A, the eight simplified synthetic analogues **8**–**15** (Figure 2) have also been synthesized. They were conceived as “functional-analogues” of smenamide A, incorporating some of the potential activity-determining structural features of the natural product. They were easily prepared thanks to the flexible nature of the previously developed synthetic route, with the aim of probing the importance of the main structural features of the smenamides, that is, the pyrrolinone, chlorovinyl and *N*-methylacetamido functional groups. In this paper, we illustrate a case-example of the application of this strategy to the design and study of functional-analogues of complex natural lead compounds.

## 2. Results and Discussion

### 2.1. Compounds ***7**–**15***

16-*epi*-smenamide A (**7**, Figure 2) is the C-16 epimer of the natural smenamide A (**1**, Figure 1). It was synthesized starting from *S*-citronellene using the chiral protocol previously reported. 16-*epi*-smenamide A was tested on SKM-M1 and RPMI-8226 cells, two MM cell lines, showing it to be able to reduce cell viability in a dose-dependent way at nanomolar concentrations (see Section 2.2). We demonstrated that 16-*epi*-smenamide A, despite possessing the opposite configuration at C-16, retains the potent antiproliferative activity shown by the natural compound, smenamide-A, thus suggesting that this configuration does not affect the nature of its activity. Therefore, as a working hypothesis for the design of simplified analogues of 16-*epi*-smenamide A (**7**), this compound was hypothetically disconnected into two main building blocks, corresponding to the polyketide and the peptide moieties. To probe the importance of the main structural features of smenamides, eight “functional-analogues” of 16-*epi*-smenamide-A were prepared. In particular, the truncated compound **8**, retaining the C1–C18 portion of smenamide A, was synthesized to investigate the role of the pyrrolinone moiety. Compounds **9**–**12**, in turn, represent the simplified C15–C27 polyketide portion and retain only the chlorovinyl and *N*-methylacetamide functional groups. They also served to investigate the role of the geometric isomerism around the C20/C21 double bond. The modulation of the polarity within the 9/11 and 10/12 pairs was achieved by acetylation. Ester **13**, only lacking the pyrrolinone moiety, was prepared to simulate the entire polyketide portion, while compound **14** and its acetyl-derivative **15** allowed us to investigate the role of the chlorine atom. In fact, it is well known that the presence of halogens in natural products is important for the modulation of the biological activity [11,12], as previously reported.

**Figure 3:**
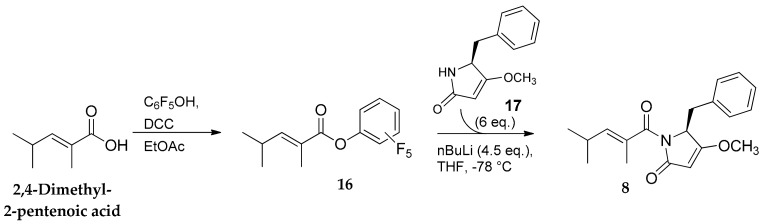
Preparation of pyrrolinone derivative **8**.

Thus, the activation of 2,4-dimethyl-2-pentenoic acid as the pentafluorophenylester (**16**) (Figure 3) and its subsequent coupling with the previously synthesized pyrrolinone subunit **17** [8], afforded compound **8** in an 85% yield.

**Figure 4:**
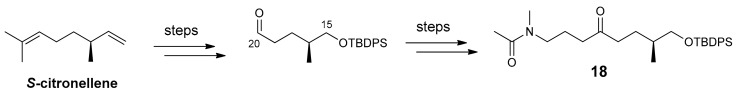
Synthesis of the ketone intermediate **18**.

**Figure 5:**
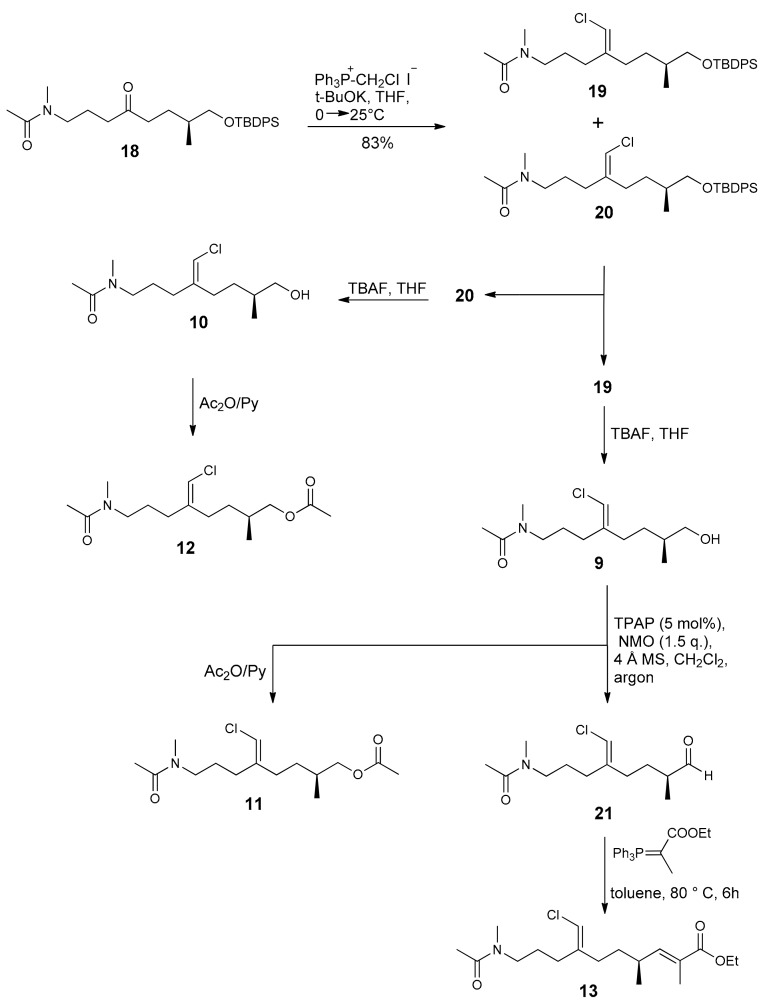
Preparation of compounds **9**–**13**.

Ketone **18** (Figure 4) is a versatile intermediate to access 16-*epi*-smenamide analogues. It was easily prepared from commercially available *S*-citronellene, as depicted in Figure 4, and used as the starting material to obtain the seven analogues **9**–**15** by the introduction of the chlorovinyl, methylene and α,β-unsaturated ethyl ester functionalities (Figure 5). Thus, the Wittig olefination of **18** gave the two isomeric chlorovinyl derivatives **19** and **20** in a 3:2 ratio in favor of **19**, which could be separated by column chromatography.

Deprotection of both **19** and **20** with tetrabutylammonium fluoride (TBAF) in tetrahydrofuran (THF) afforded alcohols **9** and **10**, respectively, whose acetylation with Ac_2_O/pyridine gave the corresponding acetyl derivatives **11** and **12**, respectively. In order to introduce the α,β-unsaturated ethyl ester function, the oxidation of **9** was accomplished with the Ley-Griffith method [tetrapropylammonium perruthenate (TPAP) (cat)/N-Methylmorpholine N-oxide (NMO)] [13,14,15] to give aldehyde **21** that was used in the subsequent Wittig reaction without further purification. Finally, the reaction with Ph_3_P=CH(Me)-CO_2_Et led to ethyl ester **13** in a 70% yield.

**Figure 6:**
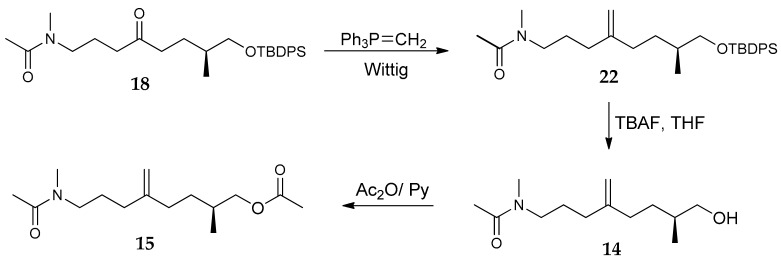
Preparation of methylene derivatives **14** and **15**.

The methylene derivatives **14** and **15** were prepared by Wittig olefination of **18** using methylenetriphenylphosphorane (Figure 6). In particular, the first obtained product **22** was deprotected with TBAF in THF to give the desired alcohol **14** whose acetylation with Ac_2_O/pyridine finally afforded the acetyl-derivative **15**.

All synthesized compounds were tested on RPMI-8226 cell lines, as described in Section 2.2. Compound **8**, lacking the great part of the polyketide moiety, was not active at all. As for the truncated polyketide compounds **9**–**15**, it was shown that only compound **13**, essentially lacking the pyrrolinone terminus, retained a certain degree of activity. In particular, a 1000-fold decreased EC50 value resulted, compared to the intact parent substance **7**. Equally, neither alcohols **9** and **10** nor the corresponding acetates **11** and **12**, not the dechlorinated analogues **14** and **15** showed significant activities. On the other hand, when the activity data of compounds **9**–**12** are compared with those of **13**, it is evident that the α,β-unsaturated ethyl ester function plays a role in the activity. In addition, even if it seems that the pyrrolinone terminus does not represent a crucial functional part of the molecule, its absence reduces the activity of **13** suggesting that it, or the entire C1–C15 unsaturated moiety, may be equally important for the full activity of smenamides, conferring rigidity to the molecule, possibly needed to exert the activity. However, these data alone do not allow us to speculate about the importance of the chlorine atom as well as of the configuration of the C20/C21 double bond on the activity.

### 2.2. In Vitro Evaluation of Activity on Multiple Myeloma Cell Lines

**Figure 7:**
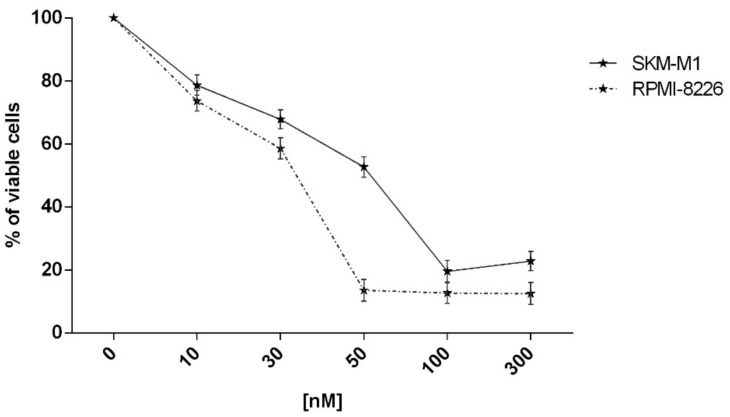
Viability of SKM-M1 and RPMI-8226 multiple myeloma (MM) cell lines was evaluated by MTS assay after treatment with compound **7** at different concentrations (10, 30, 50, 100 and 300 nM) for 48 h. Results are expressed as percent of cell viability normalized to dimethylsulfoxide (DMSO)-treated control cells. The line-graphs represent average with standard deviation (SD) from three independent experiments.

In order to study the in vitro effects of 16-*epi*-smenamide A (**7**) and its synthetic analogues **8**–**15**, MTS [3-(4,5-dimethylthiazol-2-yl)-5-(3-carboxymethoxyphenyl)-2-(4-sulfophenyl)-2H-tetrazolium, inner salt] assays were performed on SKM-M1 and RPMI-8226, MM cell lines, to evaluate their effects on cell viability. Compound **7**, tested at increasing concentrations (10–300 nM) for 48 h, was shown to reduce cell viability in both MM cell lines in a dose-dependent way (Figure 7). More than 50% of viability reduction was observed between 30 and 50 nM concentration. EC_50_ for compound **7** was calculated as 44 nM in SKM-M1 cells, and 24 nM in RPMI-8226 cells, after 48 h of treatment.

**Figure 8:**
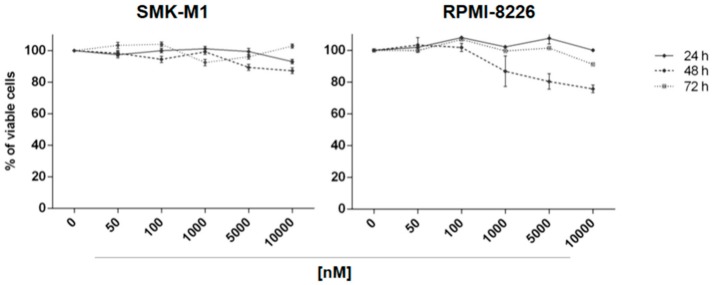
Cell viability was evaluated by MTS assay after treatment at different concentrations (50 nM, 100 nM, 1 µM, 5 µM, 10 µM) for 24, 48 and 72 h with compound **8** on SKM-M1 and RPMI-8226 cell lines. Results are expressed as percent of cell viability normalized to DMSO-treated cells. The line-graphs represent average with SD from three independent experiments.

Likewise, compound **8** was used to treat SKM-M1 and RPMI-8226 cell lines at 50 nM, 100 nM, 1 µM, 5 µM and 10 µM concentrations, for 24, 48 and 72 h. MTS assays showed that compound **8** had no effect on cell viability on SKM-M1 cell line and negligible effect on RPMI-8226 cell viability (Figure 8). For this compound, EC_50_ was not calculated.

**Figure 9:**
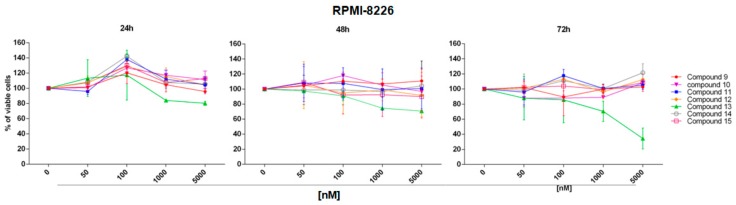
Cell viability was evaluated by MTS assay after treatment at different concentrations (50 nM, 100 nM, 1 µM, 5 µM) for 24, 48 and 72 h with compounds **9**–**15** on RPMI-8226 cell line. Results are expressed as percent of cell viability normalized to DMSO-treated cells. The line-graphs represent average with SD from three independent experiments.

Because compound **7** resulted more active on the RPMI-8226 cell line, its synthetic analogues **9**–**15** were tested on this cell line at increasing concentrations (50 nM, 100 nM, 1 µM, 5 µM) for all time points (24, 48 and 72 h). As shown in Figure 9, compound **9**–**12**, **14** and **15** have negligible effect on RPMI-8226 cell viability; while compound **13** was able to reduce cell viability reaching 80% of reduction at 5 µM, after 72 h of treatment. EC_50_ of compound **13** at 72 h was calculated as 1.1 µM.

**Figure 10:**
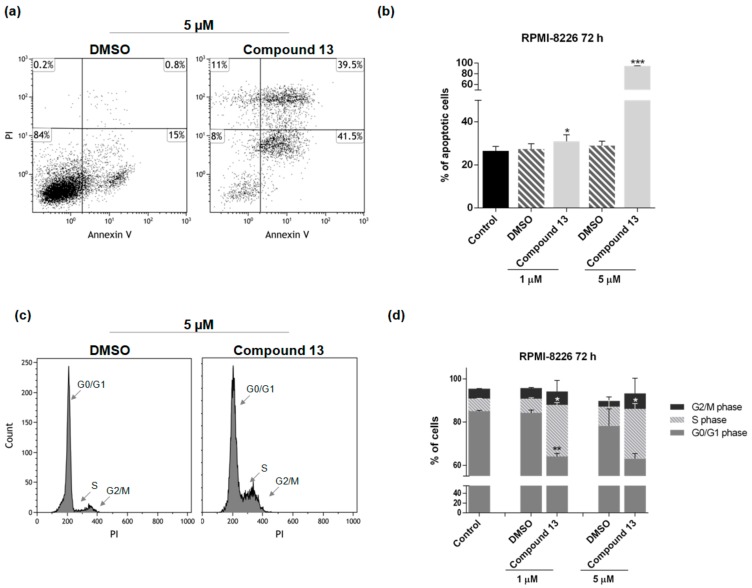
Compound **13**: cytofluorimetric evaluation of apoptosis/necrosis by the Annexin-V fluorescein isothiocyanate (FITC)/propidium iodide (PI) test (**a**,**b**) and cell cycle analysis by PI staining (**c**,**d**) on RPMI-8226 cell line, at 1 µM and 5 µM for 72 h. (**a**) Dot plots and (**c**) cell cycle histograms show a single representative experiment; (**b**,**d**) the bar-graphs represent average with S.D. (* *p* < 0.05, ** *p* < 0.01, *** *p* < 0.001).

Further investigation of the cell death mechanism was carried out using compound **13** (at 1 and 5 µM) to treat RPMI-8226 cells. Control experiments were carried out with dimethylsulfoxide (DMSO) as vehicle control, or with untreated cells. After 72 h of treatment, Annexin-V fluorescein isothiocyanate (FITC)/propidium iodide (PI) analyses were performed to evaluate whether the cytotoxic activity of compounds **13** was related to apoptosis induction. Data obtained showed that a significant increase of apoptotic cells at both concentrations occurred when cells are treated with compound **13** (5% of increase at 1 µM respect to control (* *p* < 0.05) and 66% at 5 µM (*** *p* < 0.001) (Figure 10a,b)). Moreover, compound **13** was able to significantly decrease the number of cell in G0/G1 phase and increase those in S phase at both concentrations (Figure 10c,d).

## 3. Experimental Section

### 3.1. General Experimental Procedures

All reagents and anhydrous solvents were purchased (Aldrich and Fluka) at the highest commercial quality and used without further purification. Where necessary, flame-dried and argon-charged glassware was used. The reactions were monitored using thinlayer chromatography (TLC) carried out on precoated silica gel plates (Merck 60, F254, 0.25 mm thick). Merck silica gel (Kieselgel 40, particle size 0.063–0.200 mm) was used for the column chromatography. Na_2_SO_4_ was used as a drying agent for aqueous workup. Nuclear magnetic resonance (NMR) experiments were performed using Varian Unity Inova spectrometers at 400, 500, and 700 MHz in CDCl_3_. Proton chemical shifts were referenced to the residual CHCl_3_ signal (7.26 ppm). ^13^C-NMR chemical shifts were referenced to the solvent (77.0 ppm). Abbreviations for signal coupling are as follows: s = singlet, d = doublet, t = triplet, q = quartet, m = multiplet, and b = broad. Optical rotations were measured using a JASCO P-2000 polarimeter at the sodium D line. High resolution mass spectra were recorded by infusion on a Thermo Linear Trap Quadrupole (LTQ) Orbitrap XL mass spectrometer equipped with an electrospray source in the positive mode using MeOH as the solvent.

See Supplementary Materials for all NMR spectra.

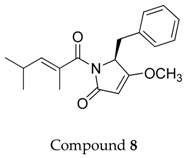



Compound **8**. To a solution of 2,4-dimethyl-2-pentenoic acid (114 mg, 0.889 mmol) in EtOAc (4.0 mL), pentafluorophenol (188.2 mg, 1.02 mmol) and DCC (210.5 mg, 1.02 mmol) were added at 0 °C. The reaction mixture was stirred for 1 h at 0 °C and 3 h at room temperature and evaporated under reduced pressure to give **16** (185.9 mg, 0.632 mmol) that was used in the next step without further purification. ^1^H-NMR: (400 MHz, CDCl_3_): δ 6.90 (1H, d, *J*=9.75), 2.8–2.6 (1H, m), 1.95 (3H, s), 1.07 (6H, d, *J* = 6.6).

To a stirred solution of pyrrolinone **17** (126.6 mg, 0.624 mmol) [8] in THF (5.0 mL), nBuLi (0.390 mL, 0.632 mmol, 1.6 M soln in hexane) was added dropwise at −78 °C. After 15 min, a solution of pentafluorophenyl ester **16** (183.45 mg, 0.624 mmol) in THF (0.1 mL) was added via syringe. After 2 h, the reaction was quenched with a saturated aqueous NH_4_Cl solution (5 mL) and extracted with EtOAc (3 × 15 mL). The organic phase was washed with water (15 mL) and brine (15 mL), dried, and concentrated in vacuo. The crude was purified by preparative TLC (CHCl_3_/CH_3_OH, 98:2) to give **8** (166.2 mg, 0.530 mmol, 85%) as colourless oil. ${\left[\alpha \right]}_{D}^{20}$ = +22.1 (c = 10, CHCl_3_); ^1^H-NMR: (400 MHz, CDCl_3_): δ 7.23–7.17 (3H, m, ArH), 7.0–6.9 (2H, m, ArH), 5.62 (1H, d, *J* = 9.47), 5.01–4.96 (1H, m), 4.84 (1H, s), 3.87 (3H, s, OCH_3_), 3.39 (1H, dd, *J* = 14.1, 5.4, H_a_-7), 3.15 (1H, dd, *J* = 14.1, 2.0, H_b_-7), 2.68–2.54 (1H, m), 1.8 (3H, s), 0.99 (6H, d, *J* = 6.5); ^13^C-NMR (100 MHz, CDCl_3_): δ 177.2, 171.3, 168.8, 145.2, 134.4, 129.8, 129.4, 128.1, 127.0, 94.8, 59.1, 58.3, 33.9, 27.4, 21.9, 21.5, 13.3; HRMS (ESI) *m/z* calcd. for C_13_H_25_ClNO_2_ [M + H]^+^ 262.1568, found 262.1566.

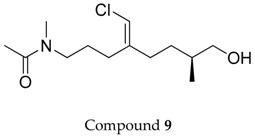



A mixture of compounds **19** and **20** was prepared as previously described [8]. Pure **19** and **20** were obtained by silica gel chromatography (hexane-EtOAc, 1:2). Deprotection of **19**, as reported [5] afforded alcohol **9** as colourless oil. ${\left[\alpha \right]}_{D}^{20}$ = −63.4 (c = 1.5, CHCl_3_); ^1^H-NMR: (400 MHz, CDCl_3_, mixture of rotamers): δ 5.86 (0.4H, s, vinyl proton), 5.82 (0.6H, s, vinyl proton), 3.46 (2H, t, *J* = 5.3), 3.42–3.24 (2H, m’s), 2.99 (1.8H, s, H_3_-27), 2.89 (1.2H, s, H_3_-27), 2.27–2.02 (7H, overlapped signals including two singlets at 2.09 and 2.07 for H_3_-26), 1.78–1.52 (4H, m), 1.30–1.15 (1H, m), 0.93, 0.91 (overall 3H, overlapped d’s, both *J* = 6.0, H_3_-17); ^13^C-NMR (100 MHz, CDCl_3_): δ 170.6, 170.4, 142.0, 141.3, 113.2, 112.6, 67.8, 67.7, 50.5, 47.3, 36.1, 35.2, 33.2, 32.3, 32.2, 31.1, 31.0, 27.4, 27.3, 25.8, 24.6, 21.9, 21.2, 16.44, 16.38; HRMS (ESI) *m/z* calcd. for C_13_H_25_ClNO_2_ [M + H]^+^ 262.1568, found 262.1566.

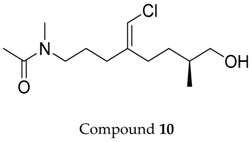



To a solution of **20** (3.9 mg, 0.008 mmol) in THF (0.6 mL), TBAF (0.012 mL, 0.012 mmol, 1.0 M solution in THF) was added at 0 °C. The reaction mixture was allowed to reach room temperature and stirred for 1 h. Then, the reaction was quenched with a satd. aq. solution of NH_4_Cl (0.5 mL). The phases were separated, and the aqueous layer was extracted with EtOAc (3 × 3 mL). The combined organic phases were dried and evaporated in vacuo. The crude was subjected to High Performance Liquid Chromatography (HPLC) separation [column Ascentis Si (Supelco), 25 cm × 4.6 mm, 5 µm; eluent: *n*-hexane/isopropanol 7:3, flow rate 1 mLmin^−1^] to give alcohol **10** (1.0 mg, 48%, t_R_ = 14.5 min) as colourless oil. ${\left[\alpha \right]}_{D}^{20}$ = +12.1 (c = 0.13; CHCl_3_); ^1^H-NMR (400 MHz, CDCl_3_, mixture of rotamers): δ 5.83 (0.4H, s, vinyl proton), 5.81 (0.6H, s, vinyl proton), 3.50 (2H, bt, *J* = 5.7), 3.34, 3.26 (1H each, both t, *J* = 7.5, H_2_-24), 2.98 (1.8H, s, H_3_-27), 2.92 (1.2H, s, H_3_-27), 2.31–2.17 (2H, m), 2.11–2.05 (5H, overlapped signals including a singlet at 2.08 for H_3_-26); 1.75–1.53 (4H, overlapped multiplets); 1.29–1.19 (1H, m), 0.98, 0.96 (overall 3H, overlapped doublets, both *J* = 6.1, H_3_-17); ^13^C-NMR (100 MHz, CDCl_3_): δ 170.7, 170.5, 142.0, 141.4, 113.0, 112.5, 67.9, 50.2, 47.4, 36.25, 36.0, 35.7, 35.6, 33.2, 32.2, 31.7, 30.33, 30.28, 27.6, 27.5, 26.2, 25.2, 22.0, 16.4; HRMS (ESI) *m/z* calcd. for C_13_H_25_ClNO_2_ [M + H]^+^ 262.1568; found 262.1566.

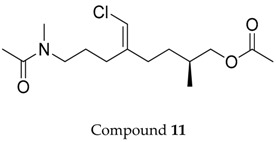



To a stirred solution of alcohol **9** (1.4 mg, 0.005 mmol) in pyridine (0.6 mL), excess acetic anhydride (0.4 mL) was added at rt. After 2 h the reaction mixture was evaporated under reduced pressure. The crude was subjected to HPLC separation [column Ascentis Si (Supelco), 25 cm × 4.6 mm, 5 µm; eluent: *n*-hexane/isopropanol 75:25, flow rate 1 mLmin^−1^] to give acetyl derivative **11** as a colourless oil (1.5 mg, 0.0047 mmol, 95%). ${\left[\alpha \right]}_{D}^{20}$ = +5.1 (c = 0.12, CHCl_3_); ^1^H-NMR: (400 MHz, CDCl_3_, mixture of rotamers): δ 5.87 (0.4H, s, vinyl proton), 5.82 (0.6H, s, vinyl proton), 3.98–3.85 (2H, m), 3.39 (1.2H, t, *J* = 6.7, H_2_-24), 3.29 (0.8H, t, *J* = 6.7, H_2_-24), 3.00 (1.8H, s, H_3_-27), 2.93 (1.2H, s, H_3_-27), 2.27–2.03 (10H, overlapped signals including singlets at 2.10, 2.09 and 2.07 for acetates), 1.80–1.54 (4H, m), 1.57–1.47 (1H, m), 1.31–1.21 (1H, m), 0.95, 0.93 (overall 3H, overlapped d’s, both *J* = 6.0, H_3_-17); ^13^C-NMR (100 MHz, CDCl_3_): δ 141.9, 141.7, 141.3, 141.0, 113.5, 112.7, 68.92, 68.83, 50.5, 47.2, 36.0, 33.2, 32.12, 32.11, 32.09, 32.08, 31.31, 31.29, 31.27, 31.26, 27.45, 27.40, 27.38, 25.8, 24.7, 21.2, 20.9, 16.7; HRMS (ESI) *m/z* calcd. for C_15_H_27_ClNO_3_ [M + H]^+^ 304.1674, found 304.1669.

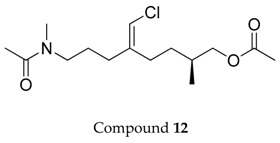



To a stirred solution of alcohol **10** (1.2 mg, 0.004 mmol) in pyridine (0.5 mL), excess acetic anhydride (0.4 mL) was added at room temperature. After 2 h the reaction mixture was evaporated under reduced pressure. The crude was subjected to HPLC separation [column Ascentis Si (Supelco), 25 cm × 4.6 mm, 5 µm; eluent: *n*-hexane/isopropanol 75:25, flow rate 1 mLmin^−1^] to give acetyl derivative **12** as colourless oil (1.0 mg, 0.003 mmol, 75%). ${\left[\alpha \right]}_{D}^{20}$ = +12.88 (c = 0.06; CHCl_3_); ^1^H-NMR: (500 MHz, CDCl_3_, mixture of rotamers): δ 5.83 (0.4H, s, vinyl proton), 5.82 (0.6H, s, vinyl proton), 3.99–3.88 (2H, m), 3.34 (1.2H, t, *J* = 7.6, H_2_-24), 3.26 (0.8H, t, *J* = 7.6, H_2_-24), 2.98 (1.8H, s, H_3_-27), 2.91 (1.2H, s, H_3_-27), 2.27–2.20 (3H, m,), 2.10–2.03 (7H, overlapped signals including singlets at 2.08, 2.07 and 2.06 for acetates), 1.85–1.45 (5H, m), 1.32–1.23 (1H, m), 0.99, 0.98 (overall 3H, overlapped d’s, both *J* = 6.0, H_3_-17); ^13^C-NMR (100 MHz, CDCl_3_): δ 171.4, 171.3, 170.6, 141.7, 141.0, 113.24, 112.7, 112.6, 69.0, 68.9, 50.2, 47.2, 36.2, 33.2, 32.44, 32.40, 31.7, 30.5, 27.5, 27.4, 26.1, 25.2, 22.0, 21.1 16.7; HRMS (ESI) *m/z* calcd. for C_15_H_27_ClNO_3_ [M + H]^+^ 304.1674; found 304.1671.

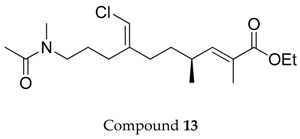



Compound **13** was prepared from alcohol **9** as previously described [8]. ${\left[\alpha \right]}_{D}^{20}$ = +127.4 (c = 0.5, CHCl_3_); IR (neat) ν_max_: 2957, 2927, 2858, 1707, 1651, 1596, 1459, 1424, 1373, 1262, 1122 cm^−1^; ^1^H-NMR (400 MHz, CDCl_3_, mixture of rotamers): δ 6.49 (1H, d, *J* = 10.1, H-15), 5.82 (0.5H, s, vinyl proton), 5.76 (0.5H, s, vinyl proton), 4.18 (2H, q, *J* = 7.0, OCH2CH3), 3.37, 3.27 (1H each, both t, *J* = 7.6, H2-24), 2.99 (1.5H, s, H_3_-27), 2.91 (1.5H, s, H_3_-27), 2.46 (1H, m, H-16), 2.18 (2H, m), 2.09 (1.5H, s, H_3_-26), 2.08 (1.5H, s, H_3_-26), 2.01 (2H, t, *J* = 8.6), 1.83 (1.5H, d, *J* = 1.2, H3-14), 1.82 (1.5H, d, *J* = 1.2, H3-14), 1.30 (3H, t, *J* = 7.0, OCH_2_CH_3_), 1.02 (1.5H, d, *J* = 6.6, H_3_-17), 1.00 (1.5H, d, *J* = 6.6, H_3_-17); ^13^C-NMR (100 MHz, CDCl_3_) δ 170.5, 170.3, 168.3, 168.2, 146.9, 146.6, 141.6, 140.8, 132.1, 132.0, 131.94, 131.91, 128.5, 128.4, 127.2, 127.0, 113.4, 112.7, 60.6, 60.5, 50.4, 47.1, 36.0, 34.7, 34.6, 33.1, 32.7, 27.4, 27.3, 25.7, 24.6, 21.9, 21.3, 20.01, 19.98, 14.3, 12.63, 12.61; HRMS (ESI) *m*/*z* calcd. for C_18_H_30_ClNNaO_3_ [M + Na]^+^ 366.1812; found 366.1802.

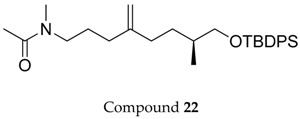



To a stirred suspension of methylenetriphenylphosphorane (6.6 mg, 0.024 mmol) in THF (0.5 mL), nBuLi (0.015 mL, 0.024 mmol, 1.6 M sol. in hexane) was added dropwise at 0 °C under argon. After 30 min at 0 °C, a solution of ketone 18 (5.5 mg, 0.012 mmol) in dry THF (0.3 + 0.3 mL rinse) was added, and the mixture was allowed to reach room temperature. After 4 h, the reaction was quenched with a saturated aqueous NH_4_Cl solution (2 mL) and extracted using Et_2_O (3× 5 mL). The organic phase was washed with brine, dried, and evaporated under reduced pressure. The crude was purified by preparative TLC (chloroform/methanol 95:5) affording compound **22** colourless oil (4.5 mg, 0.096 mmol, 80%) as a colourless oil. ^1^H-NMR (400 MHz, CDCl_3_, mixture of rotamers): δ 7.66 (4H, *J* = 6.9, ArH), 7.44–7.35 (6H, m, ArH), 4.76 (0.5H, s, methylene proton), 4.72 (0.5H, s, methylene proton), 4.71 (1H, s, methylene protons), 3.53–3.44 (2H, m), 3.34, 3.23 (1H each, both t, *J* = 7.6, H_2_-24), 2.96 (1.5H, s, H_3_-27), 2.90 (1.5H, s, H_3_-27), 2.07 (3H, s, H_3_-26), 2.05–1.92 (4H, m), 1.74–1.56 (4H, m), 1.32–1.17 (1H, m), 1.05 (9H, s, C(CH_3_)_3_), 0.93 (3H, d, *J* = 6.5, H_3_-17); ^13^C-NMR (100 MHz, CDCl_3_): δ 170.4, 149.2, 148.4, 135.6, 134.0, 133.9, 129.52, 129.48, 109.5, 108.9, 68.8, 68.7, 50.5, 47.4, 36.1, 35.4, 33.45, 33.38, 33.2, 32.8, 31.2, 29.7, 26.9, 26.1, 25.3, 21.9, 21.2, 19.3, 16.7; HRMS (ESI) *m/z* calcd. for C_29_H_43_NO_2_Si [M + H]^+^ 466.3136; found 466.3124.

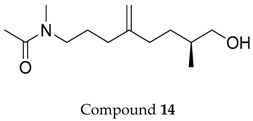



To a solution of **22** (5.4 mg, 0.012 mmol) in THF (0.8 mL), TBAF (0.017 mL, 0.017 mmol, 1.0 M solution in THF) was added at 0 °C. The reaction mixture was allowed to reach rt and stirred for 1 h. Then, the reaction was quenched with a satd. aq. solution of NH_4_Cl (1 mL). The phases were separated, and the aqueous layer was extracted with EtOAc (3 × 5 mL). The combined organic phases were dried and evaporated in vacuo. The crude was subjected to HPLC separation [column Ascentis Si (Supelco), 25 cm × 4.6 mm, 5 µm; eluent: ethyl acetate/isopropanol 9:1, flow rate 1 mLmin^−1^] to give alcohol **14** (1.9 mg, 0.008 mmol, 70%) as colourless oil. ${\left[\alpha \right]}_{D}^{20}$ = +7.24 (c = 0.07; CHCl_3_); ^1^H-NMR (400 MHz, CDCl_3_, mixture of rotamers): δ 4.79 (0.5H, s, methylene proton), 4.75 (1.5H, bs, methylene protons), 3.54–3.43 (2H, m), 3.41–3.30 (1H, m, H_2_-24), 3.27 (1H, t, *J*=7.4, H_2_-24), 2.99 (1.5H, s, H_3_-27), 2.92 (1.5H, s, H_3_-27), 2.12 1.98 (overall 7H, including singlets at 2.09 and 2.07 for H_3_-26), 1.75–1.50 (4H, m), 1.32–1.19 (1H, m), 0.95, 0.93 (overall 3H, overlapped d’s, *J* = 6.5, H_3_-17); ^13^C-NMR (100 MHz, CDCl_3_): δ 170.6, 150.8, 148.8, 148.3, 109.6, 109.2, 68.14, 68.10, 50.4, 47.5, 36.3, 35.4, 33.4, 33.2, 33.1, 33.0, 32.1, 31.08, 31.03, 29.7, 26.0, 25.1, 21.3, 16.6, 16.5; HRMS (ESI) *m/z* calcd. for C_13_H_26_NO_2_ [M + H]^+^ 228.1958; found 228.1956.

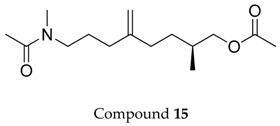



To a stirred solution of alcohol **14** (1.5 mg, 0.006 mmol) in pyridine (0.2 mL), excess acetic anhydride (0.2 mL) was added at rt. After 2 h the reaction mixture was evaporated under reduced pressure. The crude was subjected to HPLC separation [column Ascentis Si (Supelco), 25 cm × 4.6 mm, 5 µm; eluent: *n*-hexane/isopropanol 75:25, flow rate 1 mLmin^−1^] to give acetyl derivative **15** as a colourless oil (1.0 mg, 0.004 mmol, 62%). ${\left[\alpha \right]}_{D}^{20}$ = +13.63 (c = 0.07; CHCl_3_); ^1^H-NMR (400 MHz, CDCl_3_, mixture of rotamers): δ 4.78 (0.5H, s, methylene proton), 4.75 (0.5H, s, methylene proton), 4.74 (1H, s, methylene protons), 3.99–3.84 (2H, m), 3.36, 3.26 (1H each, both t, *J* = 7.6, H_2_-24), 2.98 (1.5H, s, H_3_-27), 2.92 (1.5H, s, H_3_-27), 2.12–1.96 (10H, overlapped signals including singlets at 2.09, 2.08 and 2.06 for acetates) 1.82–1.60 (4H, m), 1.34–1.22 (1H, m), 0.95, 0.94 (overall 3H, overlapped d’s, *J* = 6.5, H_3_-17); ^13^C-NMR (175 MHz, CDCl_3_): δ 170.42, 171.36, 170.6, 141.7, 141.05, 113.24, 112.6, 112,65, 69.0, 68.9, 50.2, 47.2, 36.2, 33.2, 32.43, 32.40, 31.9, 31.7, 30.5, 27.5, 27.4, 26.1, 25.2, 22.0, 21.4, 21.1, 16.7; HRMS (ESI) *m/z* calcd. for C_15_H_27_NO_3_ [M + H]^+^ 270.2063; found 270.2061.

### 3.2. Biological Activity

#### 3.2.1. Cell Lines and Chemical

Human MM cell lines, SKM-M1 and RPMI-8226, were cultured in RPMI 1640 (Gibco, Life Technologies, Carlsbad, CA, USA) supplemented with 10% fetal bovine serum (FBS. Gibco, Life Technologies, Carlsbad, CA, USA), 1% of penicillin-streptomycin (Gibco) at 37 °C and 5% CO_2_.

All chemical compounds were dissolved in DMSO (Sigma Aldrich, St. Louis, MO, USA) and diluted in FBS for cell treatments.

#### 3.2.2. Cell Viability

SKM-M1 and RPMI-8226 cell lines were seeded into 96-well plates (3 × 10^4^ cells/100 μL) and incubated with all compounds at increasing concentrations for different time points. In particular, compound **7** was used at concentrations 10–300 nM for 48 h; compound **8** at 50–10 µM for 24, 48 and 72 h; compounds **9**–**15** at 50–5 µM for 24, 48 and 72 h. Cells treated with the DMSO vehicle were used as control. Cell viability was determined using the CellTiter 96 Aqueous One Solution assay kit (MTS, Promega, Madison, WI, USA). The optical density was measured at 492 nm by plate reader (Das srl, Rome, Italy). Cellular viability was calculated as percentage of viable cells compared with DMSO control. All experiments were conducted in triplicate. EC_50_ values were obtained by GraphPad Prism (GraphPad Prism, San Diego, CA, USA).

#### 3.2.3. Functional Tests

RPMI-8226 cell line was treated with 1 μM and 5 μM of compound **13** or with DMSO vehicle or not treated for 72 h (cell density 3×10^5^ cells/mL) and used in:

- Apoptosis assay

Apoptosis of RPMI-8226 was evaluated by cytometric analysis of Annexin V and PI-stained cells using fluorescein isothiocyanate (FITC) Annexin V Apoptosis Detection kit I (Becton Dickinson, BD, Franklin, NJ, USA) [16]. Samples were prepared following the manufacturer’s instructions; stained cells were acquired using NAVIOS flow cytometer (Beckman Coulter, Brea, CA, USA) and analyzed by Kaluza software (Beckman Coulter). 10,000 events were acquired for each samples; single positive for Annexin V and double positive for Annexin V and PI cells were interpreted as signs of early and late phases of apoptosis respectively. Percent of apoptotic cells was obtained from the sum of early and late apoptosis.

- Cell cycle analysis

After treatment RPMI-8226 cells were fixed in cold ethanol 70% for 1 h, then labeled with PI (Sigma Aldrich, St. Louis, MO, USA)/RNase A (EuroClone S.p.a., Pero, MI, Italy) staining solution for 30 min. Samples were acquired by NAVIOS flow cytometer and analyzed by Kaluza software (Beckman Coulter). 10,000 events were acquired for each sample.

#### 3.2.4. Statistical Analysis

Statistical significance was determined using a paired t test by GraphPad Prism. All error bars represent the standard deviation (SD) of the average.

## 4. Conclusions

This study adds new knowledge about the antiproliferative activity and the possible role of smenamides, chlorinated peptide/polyketide substances originally isolated from the Caribbean sponge *Smenospongiaaurea*, as lead compounds in anticancer drug research. Our results have shown that the configuration at C-16 slightly affects the activity, since the 16-*epi*-analogue **7** was still active at nanomolar concentrations. Interestingly, it has been found that the truncated compound **8**, containing the pyrrolinone terminus, was not active while compound **13**, composed of the intact C12–C27 portion, retained the activity, even though its EC50 value was 1000 times smaller compared with the parent 16-*epi*-smenamide **7**. In addition, compound **13** was able to block the cell cycle at the G0/G1 phase. It is worth noting that smenothiazoles [17], biogenetically related but structurally different from smenamides, possess the same activity. This study provides the basic knowledge needed to design simplified and synthetically easily accessible analogues that could target MM cells.

## Supplementary Materials

The following are available online at http://www.mdpi.com/1660-3397/16/6/206/s1, Figure S1: ^1^H NMR spectrum of compound 16 (CDCl_3_, 400 MHz), Figure S2: ^13^C NMR spectrum of compound 16 (CDCl_3_, 100 MHz), Figure S3: ^1^H NMR spectrum of compound 8 (CDCl_3_, 400 MHz), Figure S4: ^13^C NMR spectrum of compound 8 (CDCl_3_, 100 MHz), Figure S5: ^1^H NMR spectrum of compound 10 (CDCl_3_, 400 MHz), Figure S6: ^13^C NMR spectrum of compound 10 (CDCl_3_, 100 MHz), Figure S7: ^1^H NMR spectrum of compound 11 (CDCl_3_, 400 MHz), Figure S8: ^13^C NMR spectrum of compound 11 (CDCl_3_, 100 MHz), Figure S9: ^1^H NMR spectrum of compound 12 (CDCl_3_, 400 MHz), Figure S10: ^13^C NMR spectrum of compound 12 (CDCl_3_, 100 MHz), Figure S11: ^1^H NMR spectrum of compound 22 (CDCl_3_, 400 MHz), Figure S12: ^13^C NMR spectrum of compound 22 (CDCl_3_, 100 MHz), Figure S13: ^1^H NMR spectrum of compound 14 (CDCl_3_, 400 MHz), Figure S14: ^13^C NMR spectrum of compound 14 (CDCl_3_, 100 MHz), Figure S15: ^1^H NMR spectrum of compound 15 (CDCl_3_, 400 MHz), Figure S16: ^13^C NMR spectrum of compound 15 (CDCl_3_, 100 MHz).
