# Supplementary material for: Smenamide A Analogues. Synthesis and Biological Activity on Multiple Myeloma Cells
Source: Mar Drugs. 2018 Jun 13;16(6):206. doi: 10.3390/md16060206 (PMC6025564; doi:10.3390/md16060206)
Supplement: Supplementary file 1 [file marinedrugs-16-00206-s001.pdf]

Smenamide A analogues. Synthesis and biological activity on multiple myeloma cells.

Alessia Caso [1,§], Ilaria Laurenzana [2,§], Daniela Lamorte [2], Stefania Trino [2], Germana Esposito [1], Vincenzo Piccialli [3,*], and Valeria Costantino [1*]

[1] Department of Pharmacy, University of Naples Federico II, 80131 Napoli, Italy;

[2] Laboratory of Pre-Clinical and Translational Research, IRCCS—Referral Cancer Center of Basilicata (CROB), 85028 Rionero in Vulture (PZ), Italy;

[3] Department of Chemical Sciences, University of Naples Federico II, via Cintia 4, 80126 Naples, Italy.

Supplementary Material

Figure S1: ^{1}H NMR spectrum of compound **16** ($CDCl_3$, 400 MHz).

Figure S2: ^{13}C NMR spectrum of compound **16** ($CDCl_3$, 100 MHz).

Figure S3: ^{1}H NMR spectrum of compound **8** ($CDCl_3$, 400 MHz).

Figure S4: ^{13}C NMR spectrum of compound **8** ($CDCl_3$, 100 MHz).

Figure S5: ^{1}H NMR spectrum of compound **10** ($CDCl_3$, 400 MHz).

Figure S6: ^{13}C NMR spectrum of compound **10** ($CDCl_3$, 100 MHz)

Figure S7: ^{1}H NMR spectrum of compound **11** ($CDCl_3$, 400 MHz).

Figure S8: ^{13}C NMR spectrum of compound **11** ($CDCl_3$, 100 MHz).

Figure S9: ^{1}H NMR spectrum of compound **12** ($CDCl_3$, 400 MHz).

Figure S10: ^{13}C NMR spectrum of compound **12** ($CDCl_3$, 100 MHz).

Figure S11: ^{1}H NMR spectrum of compound **22** ($CDCl_3$, 400 MHz).

Figure S12: ^{13}C NMR spectrum of compound **22** ($CDCl_3$, 100 MHz).

Figure S13: ^{1}H NMR spectrum of compound **14** ($CDCl_3$, 400 MHz).

Figure S14: ^{13}C NMR spectrum of compound **14** ($CDCl_3$, 100 MHz).

Figure S15: ^{1}H NMR spectrum of compound **15** ($CDCl_3$, 400 MHz).

Figure S16: ^{13}C NMR spectrum of compound **15** ($CDCl_3$, 100 MHz).

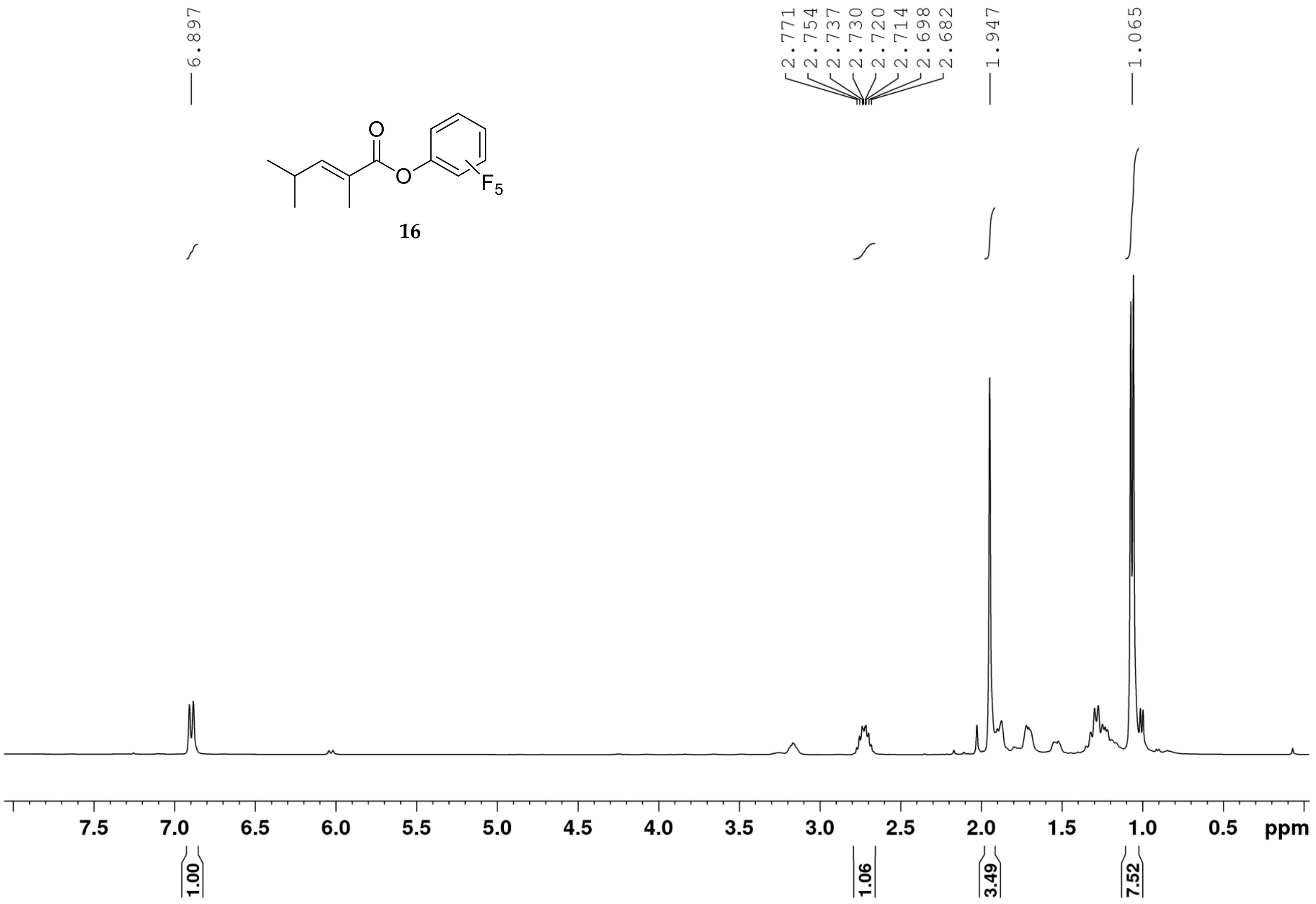

Figure S1. ^{1}H NMR spectrum of compound **16** ($CDCl_3$, 400 MHz).

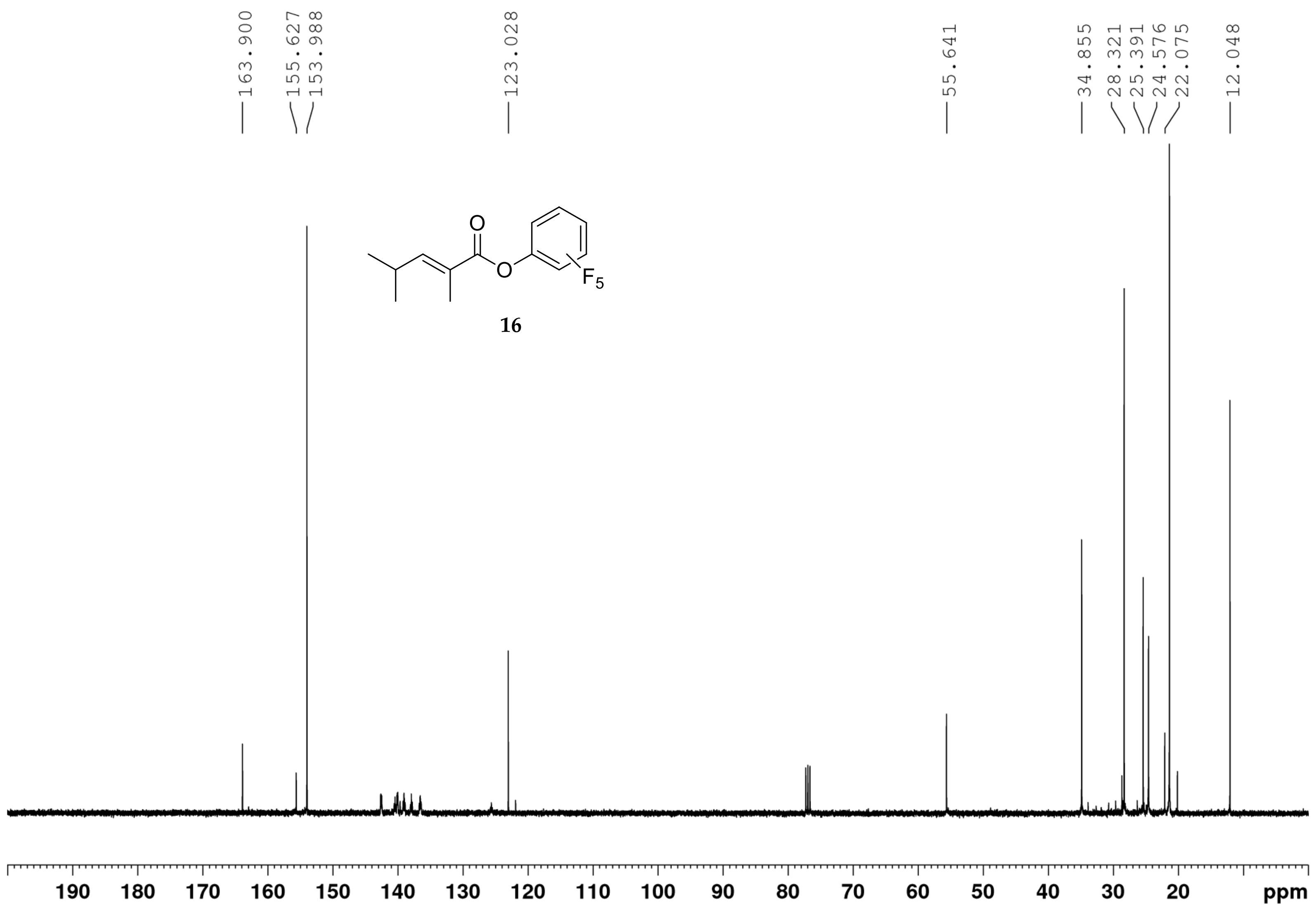

Figure S2. ^{13}C NMR spectrum of compound **16** ($CDCl_3$, 100 MHz).

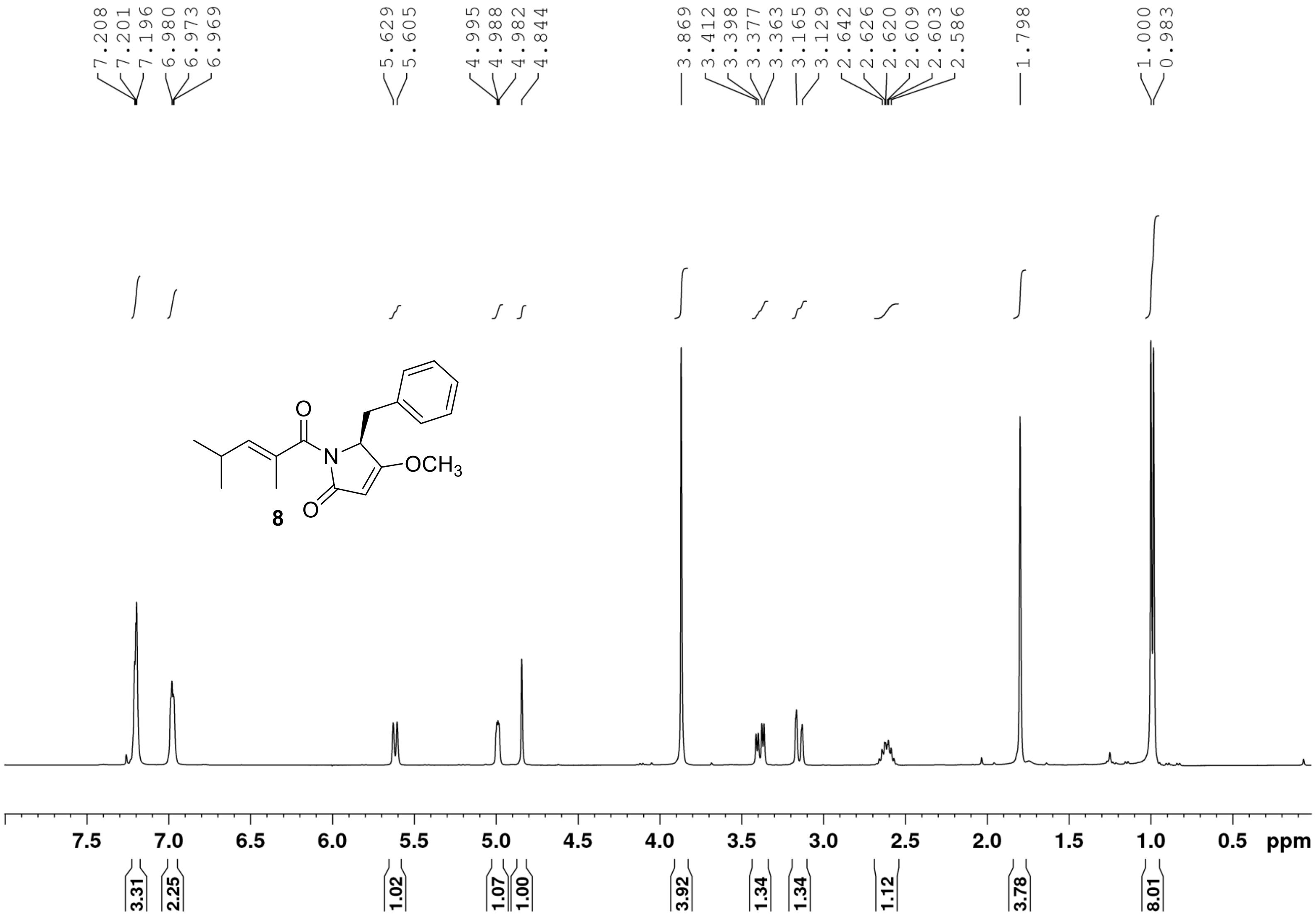

Figure S3. ^{1}H NMR spectrum of compound **8** (CDCl3, 400 MHz).

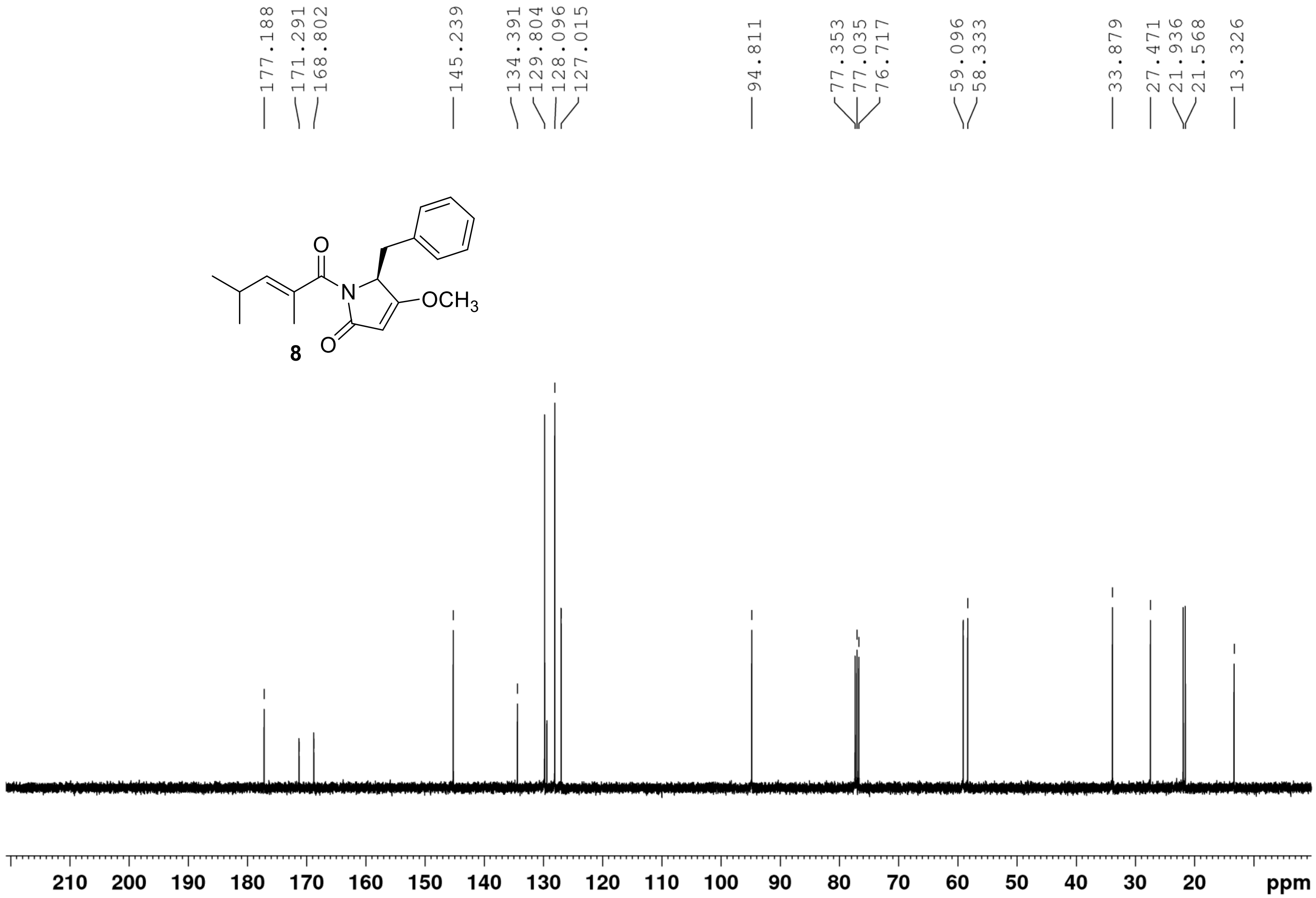

Figure S4. ^{13}C NMR spectrum of compound **8** ($CDCl_3$, 100 MHz).

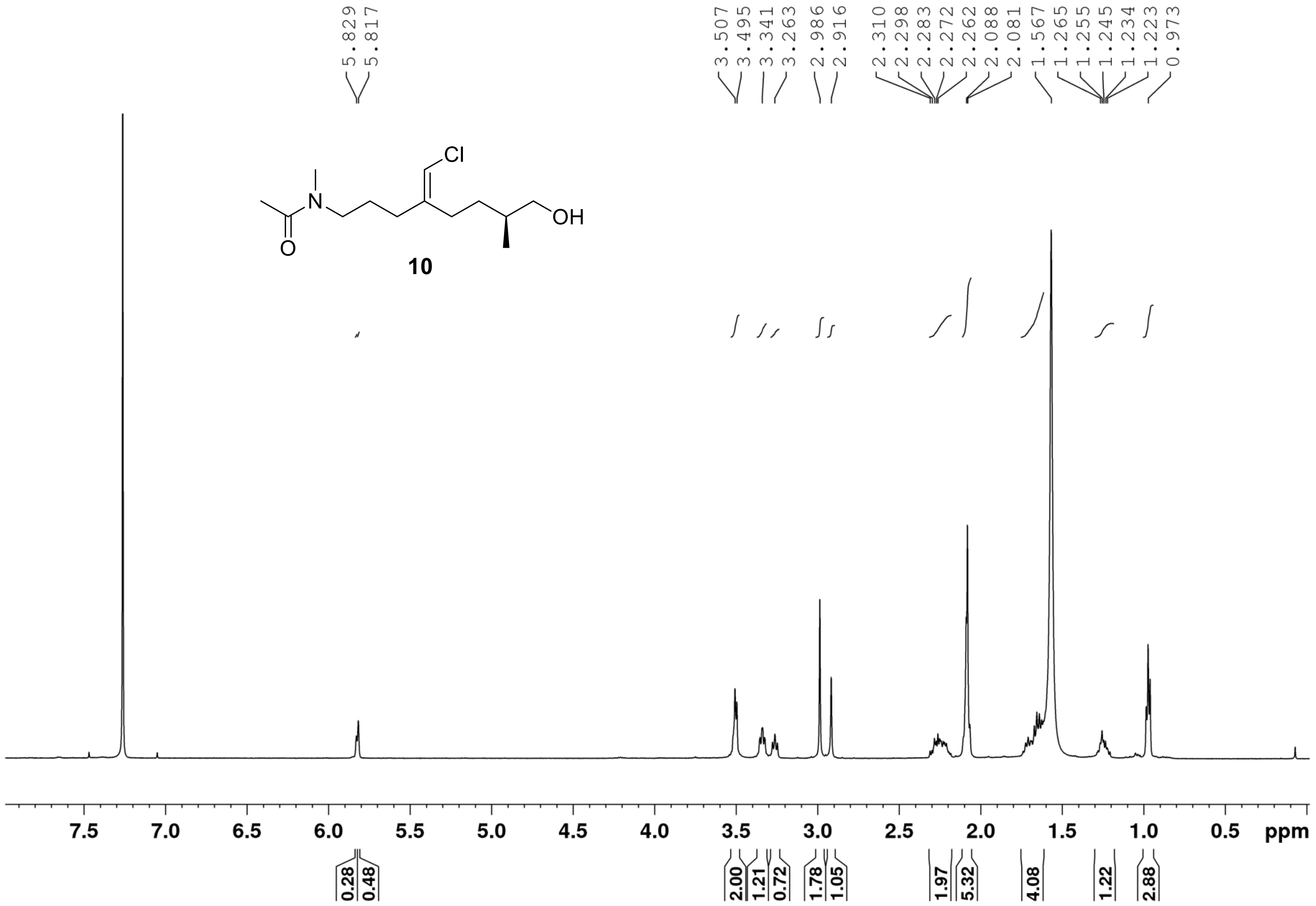

Figure S5. ^{1}H NMR spectrum of compound **10** ($CDCl_3$, 400 MHz).

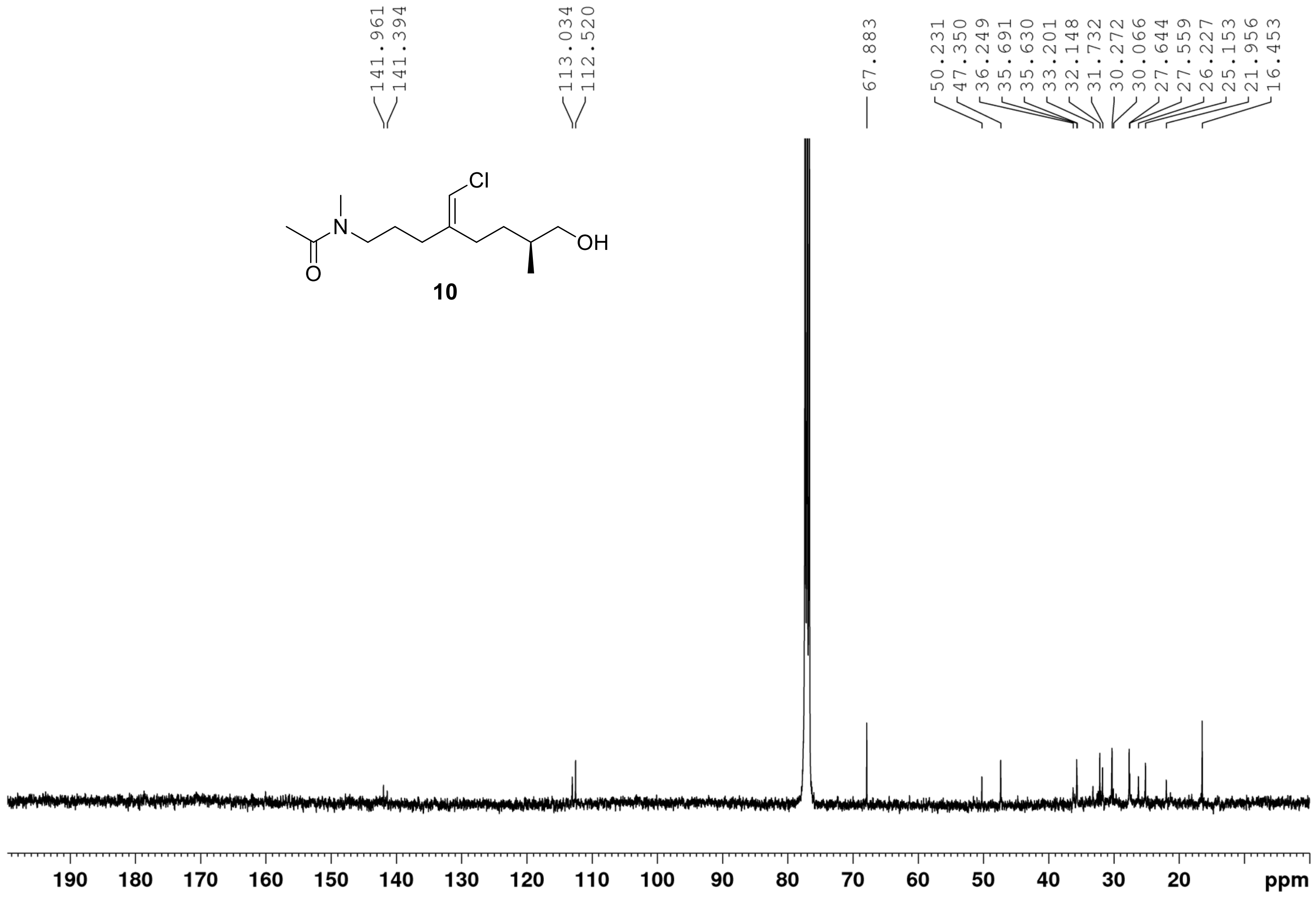

Figure S6. ^{13}C NMR spectrum of compound **10** ($CDCl_3$, 100 MHz).

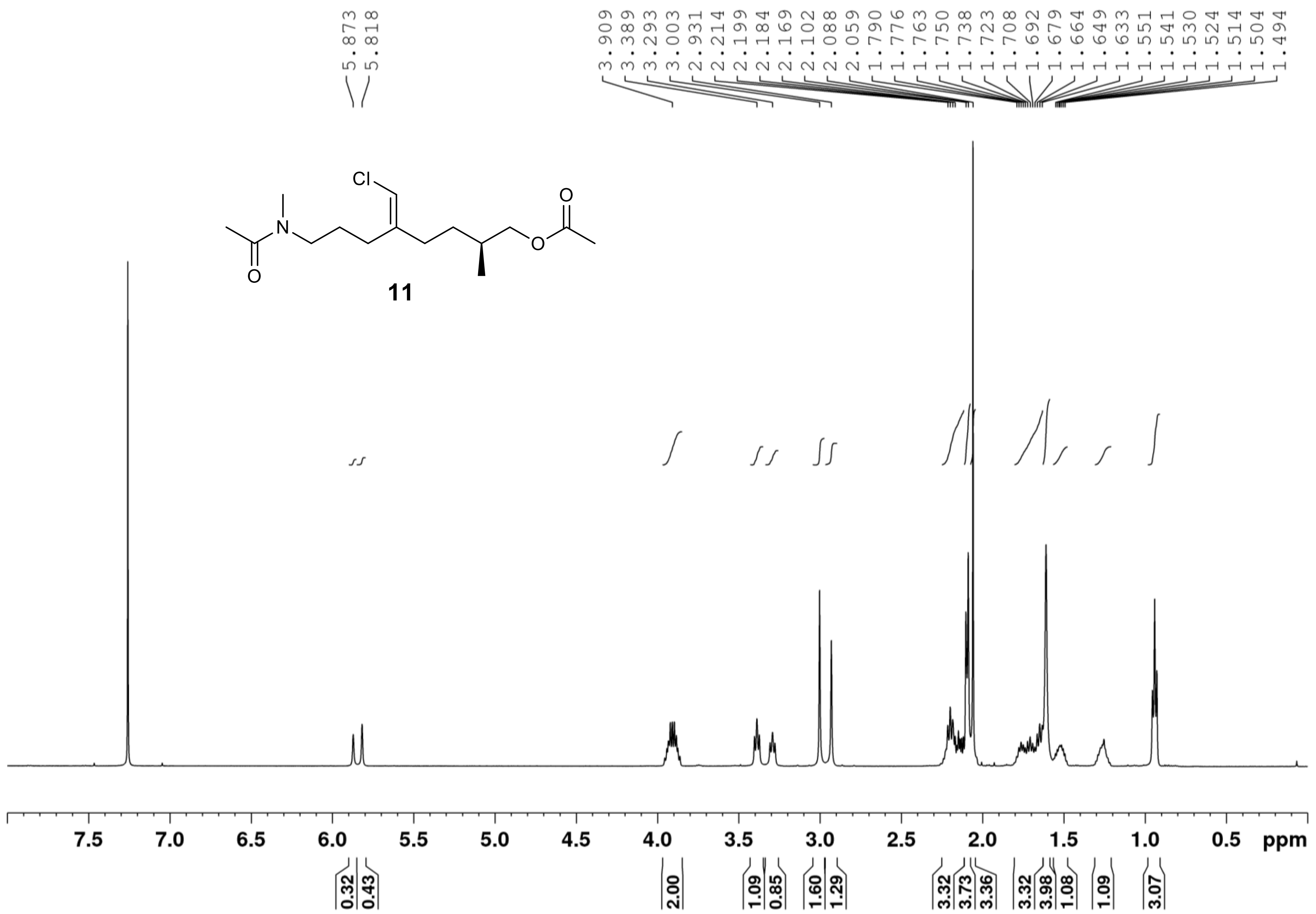

Figure S7. ^{1}H NMR spectrum of compound **11** (CDCl3, 400 MHz).

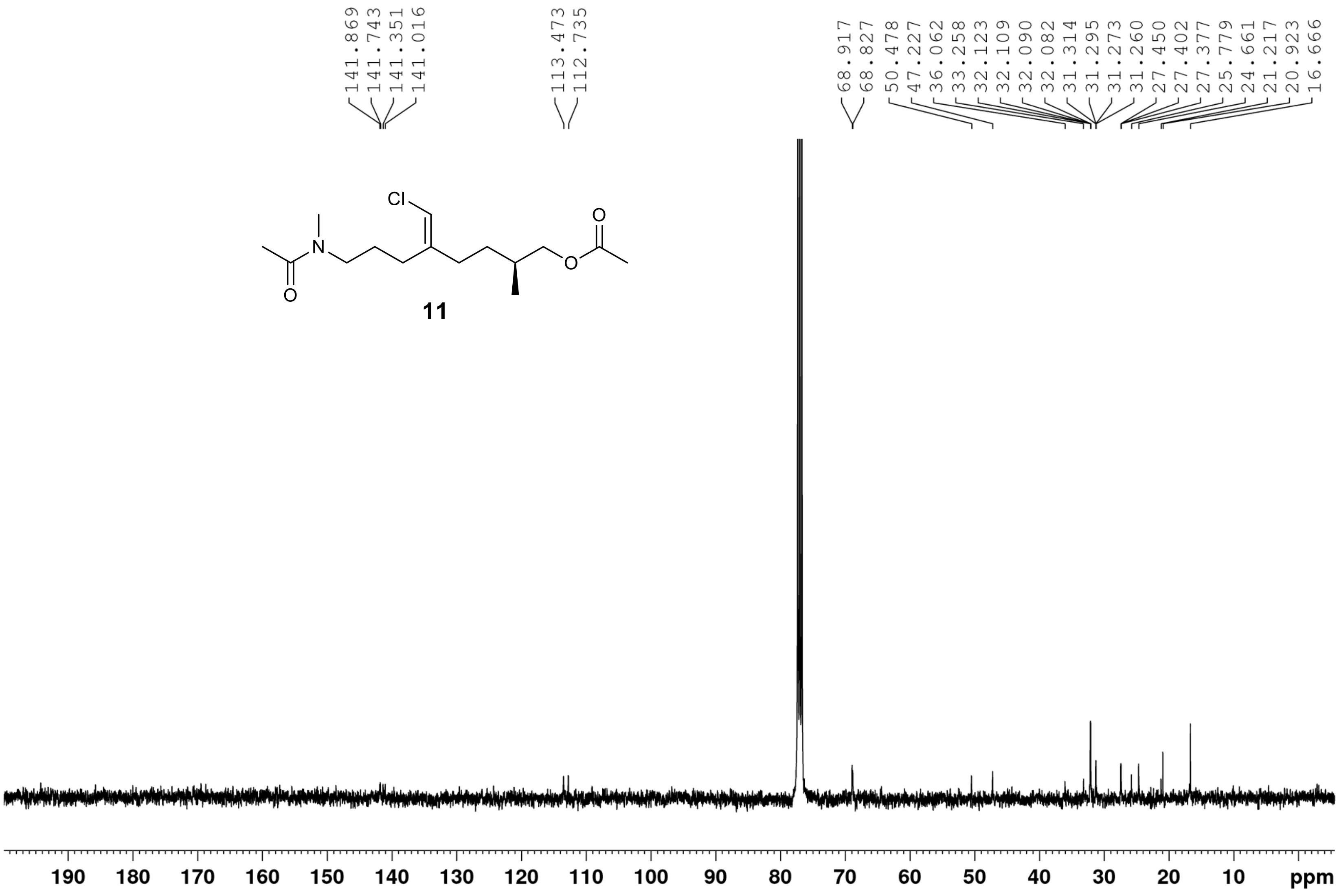

Figure S8. [13] C NMR spectrum of compound **11** (CDCl3, 100 MHz).

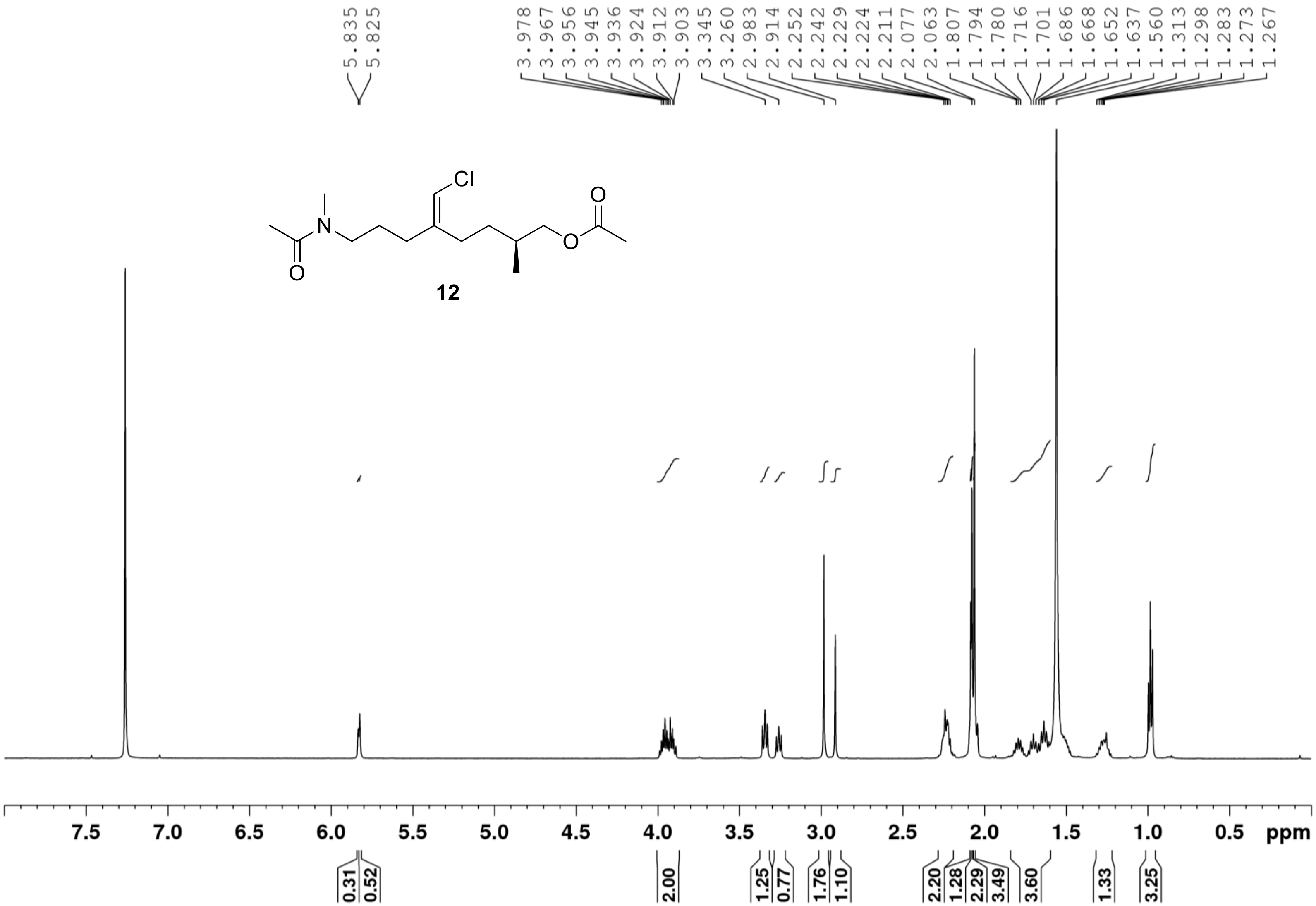

Figure S9. ^{1}H NMR spectrum of compound **12** ($CDCl_3$, 400 MHz).

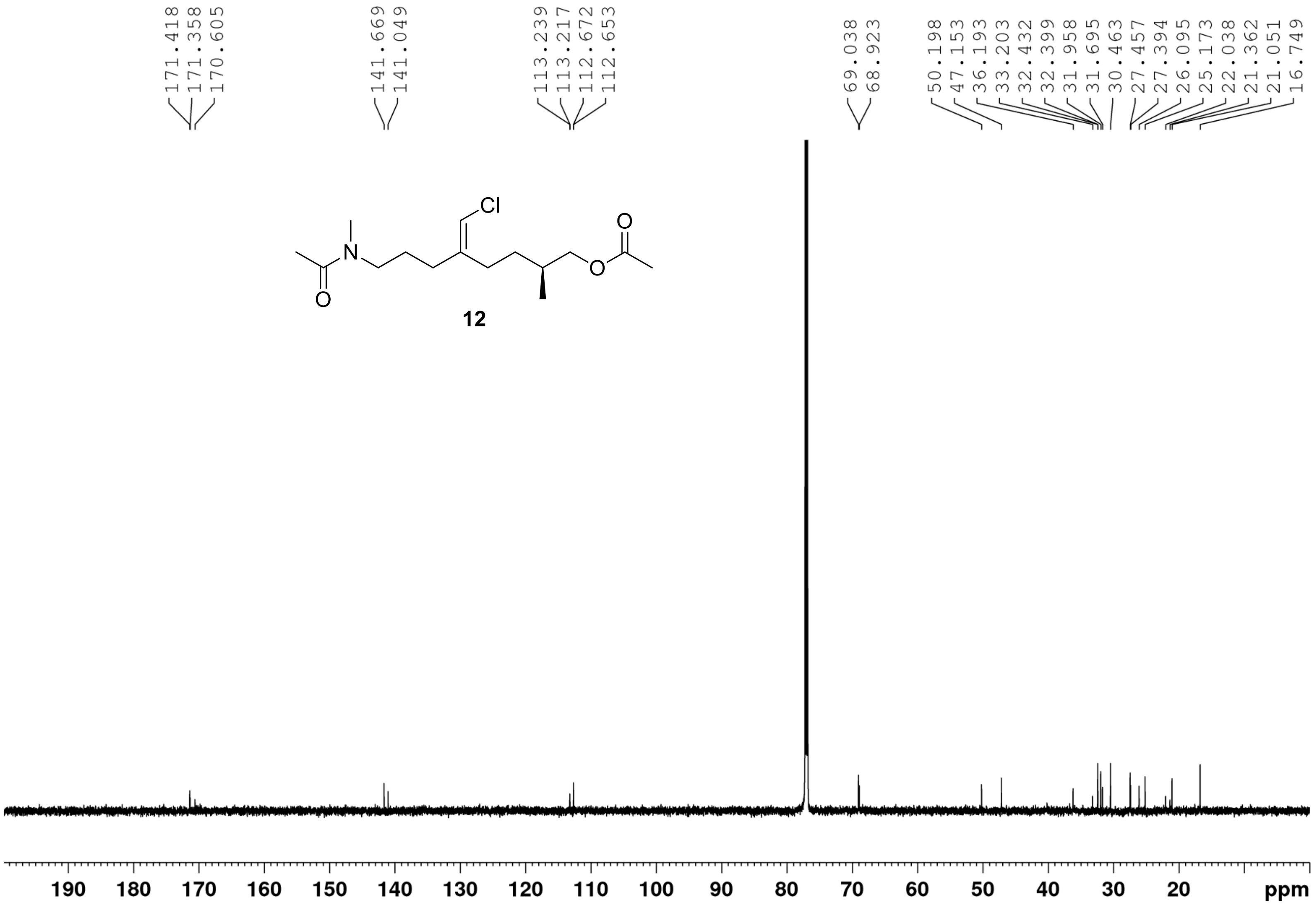

Figure S10. ^{13}C NMR spectrum of compound **12** ($CDCl_3$, 100 MHz).

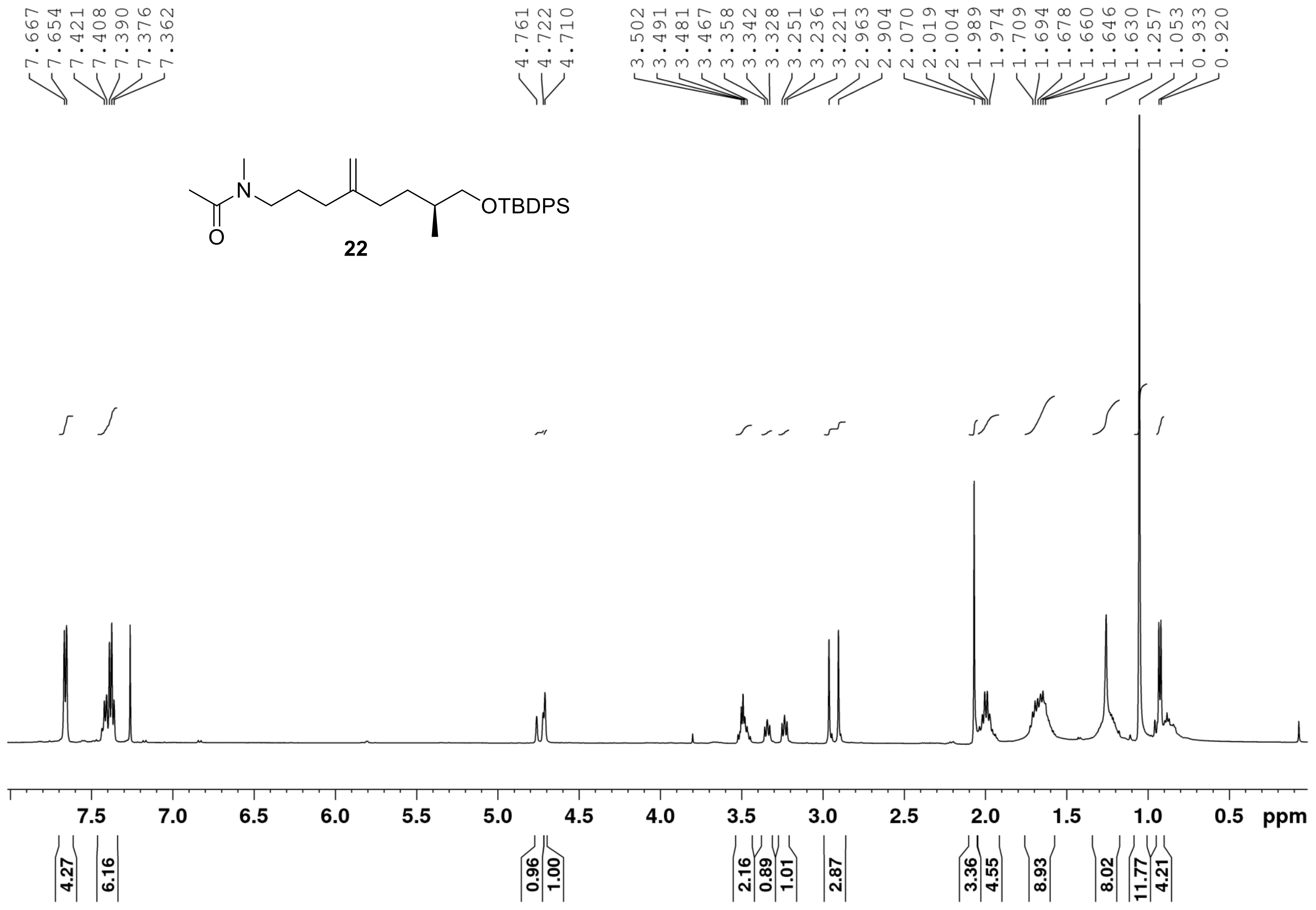

Figure S11. ^1H NMR spectrum of compound **22** ($CDCl_3$, 400 MHz).

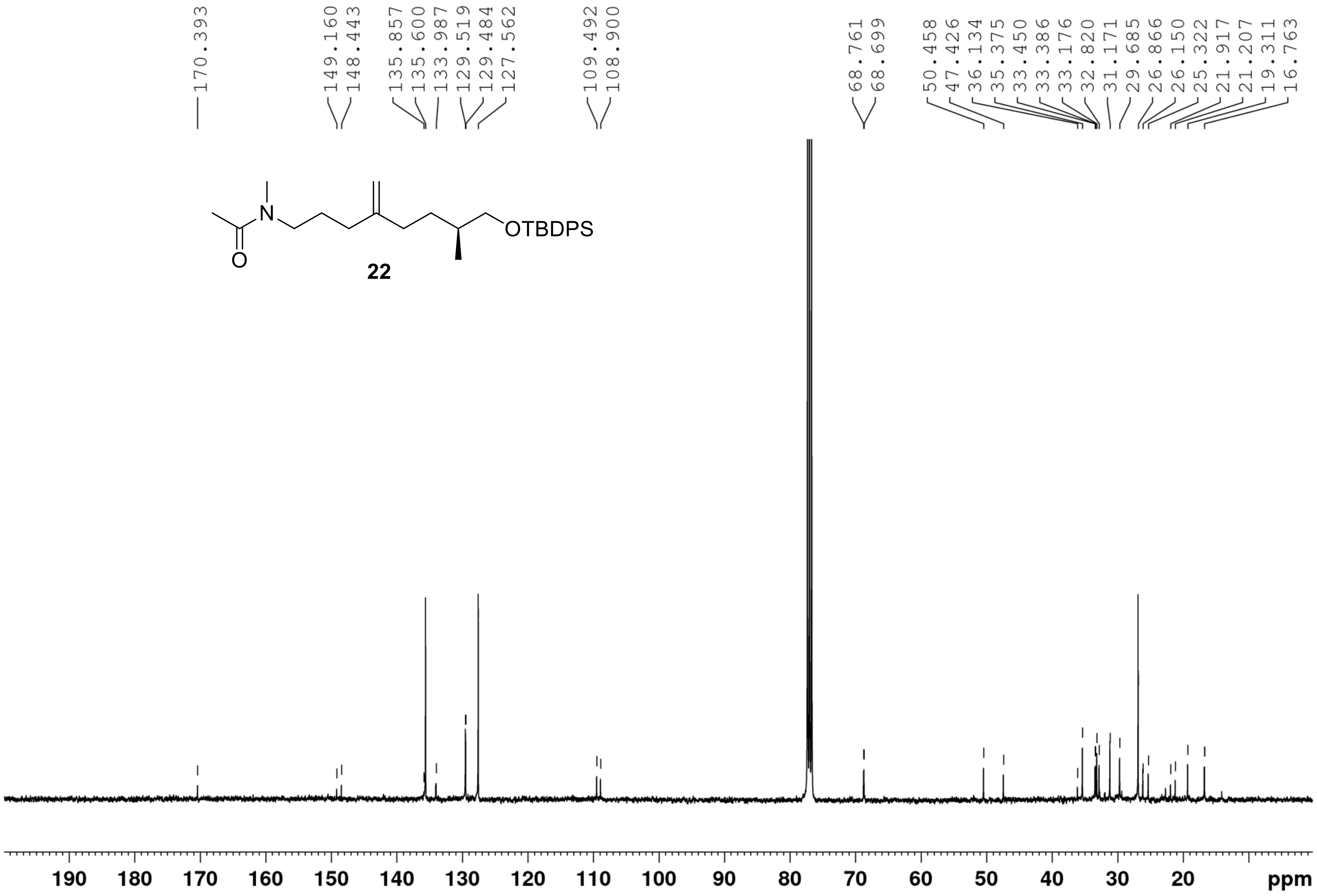

Figure S12. ^{13}C NMR spectrum of compound **22** ($CDCl_3$, 100 MHz).

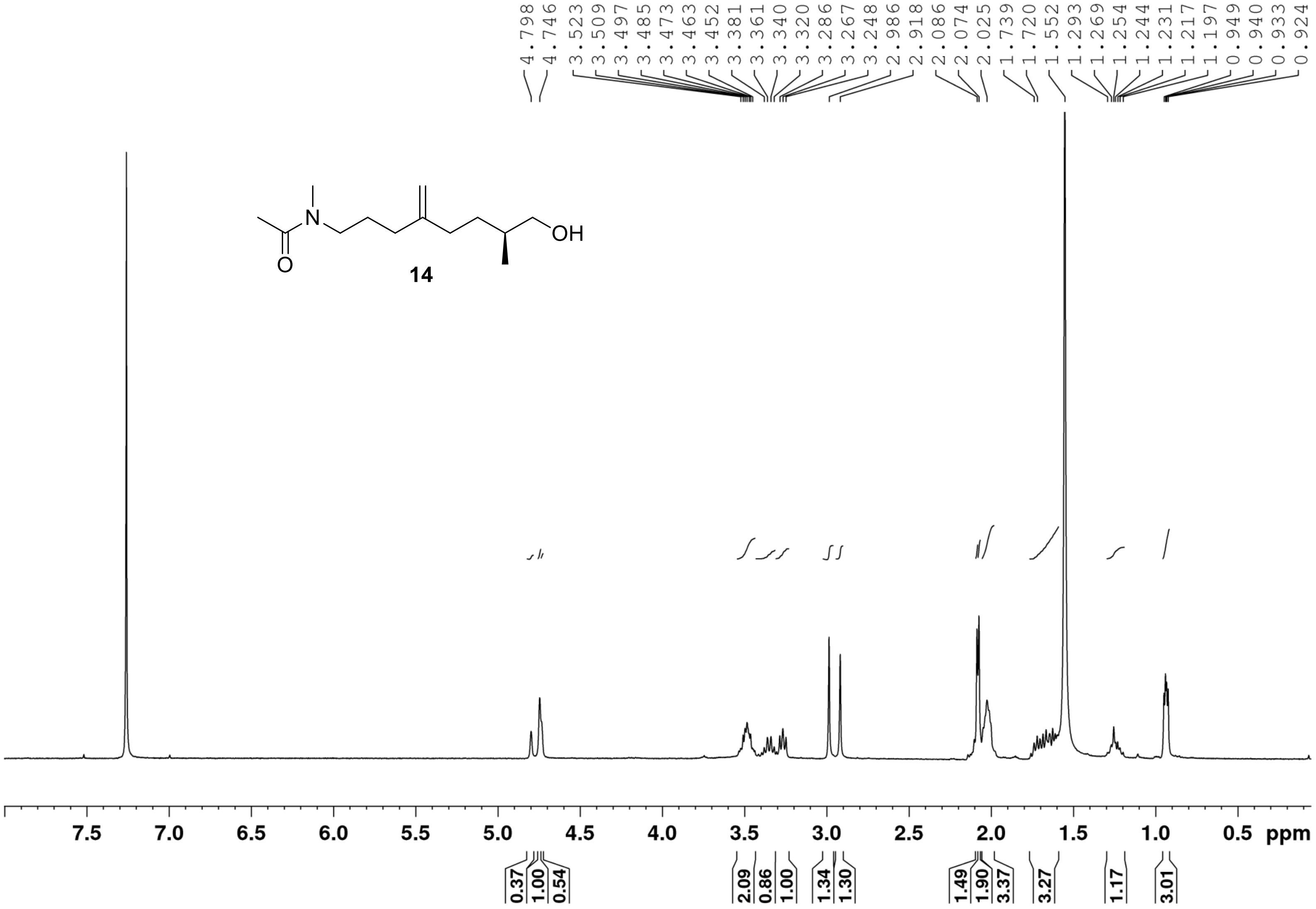

Figure S13. ^{1}H NMR spectrum of compound **14** ($CDCl_3$, 400 MHz).

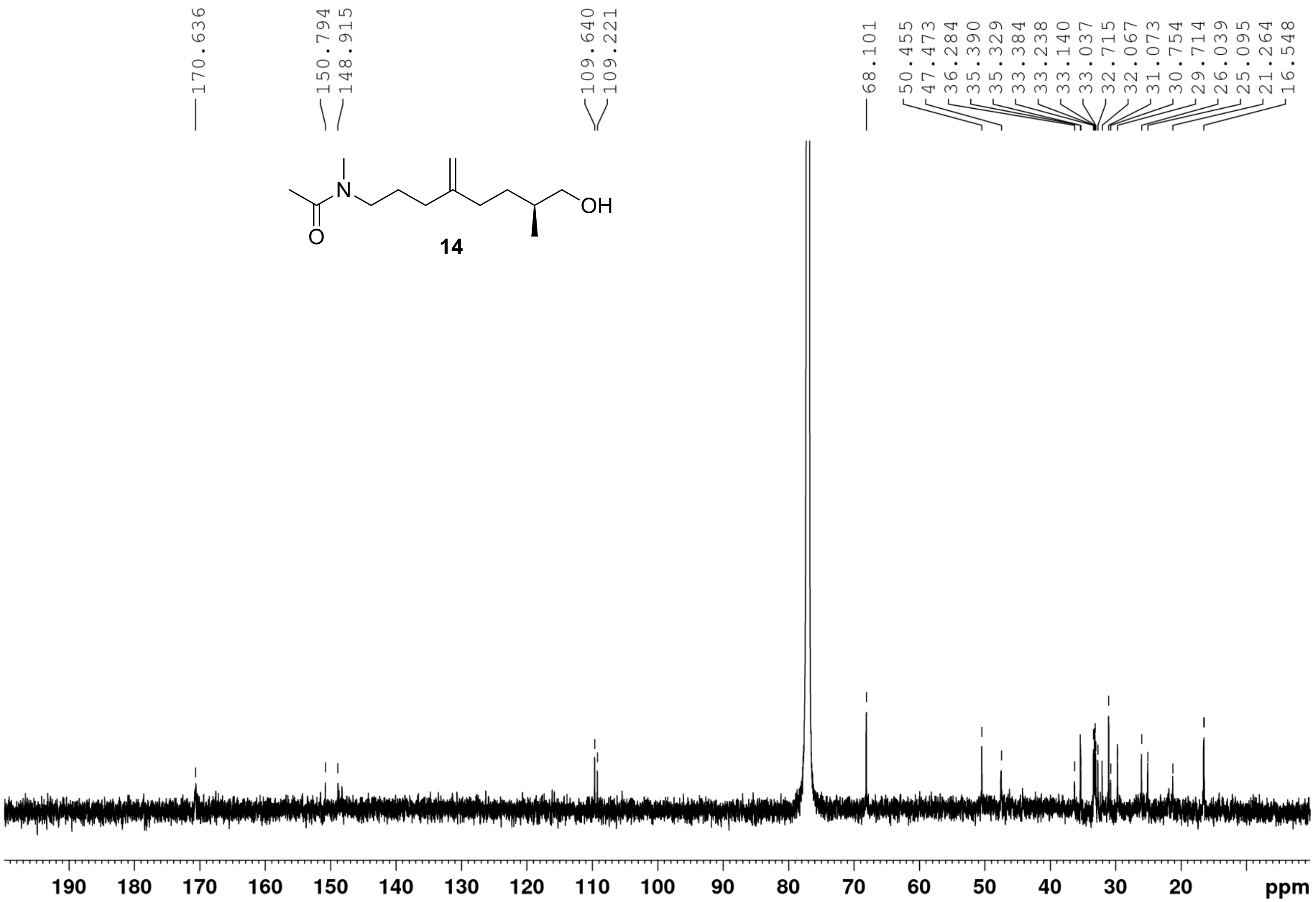

Figure S14. ^{13}C NMR spectrum of compound **14** ($CDCl_3$, 100 MHz).

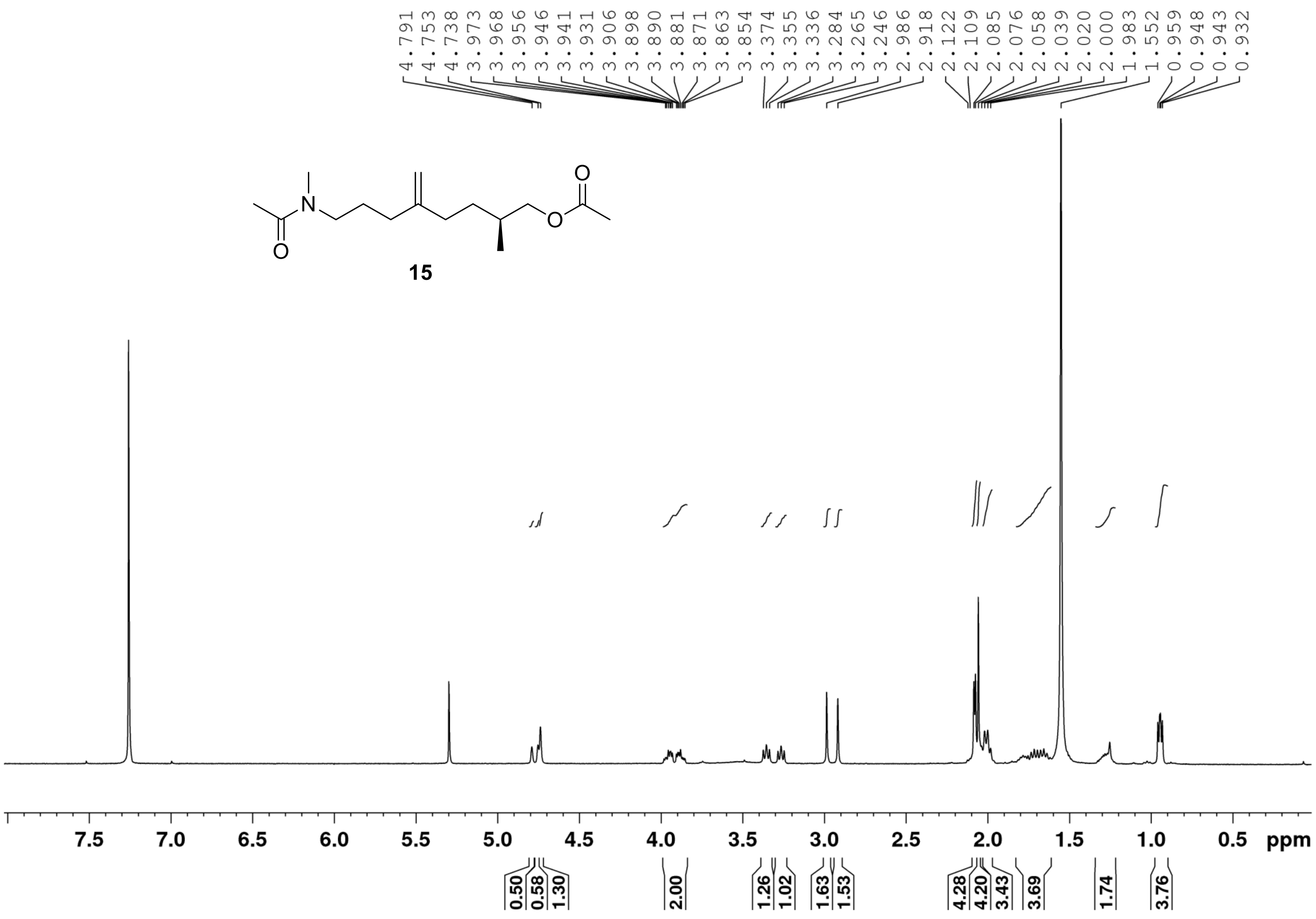

Figure S15. ^{1}H NMR spectrum of compound **15** ($CDCl_3$, 400 MHz).

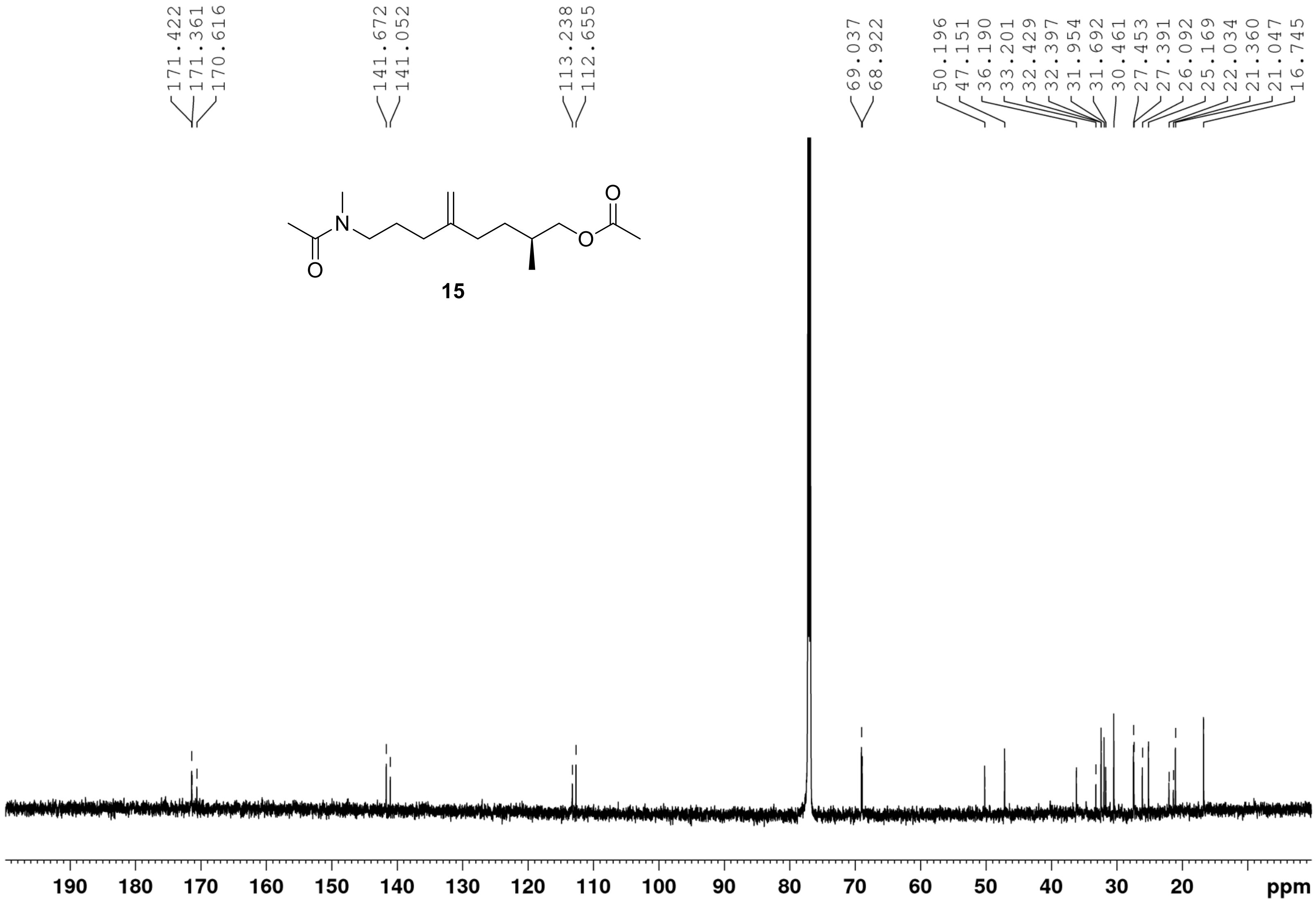

Figure S16. ^{13}C NMR spectrum of compound **15** ($CDCl_3$, 100 MHz).
